# Notch/NF-κB crosstalk signaling controls SASP gene expression during brain aging

**DOI:** 10.1093/nar/gkag1110

**Published:** 2026-11-05

**Authors:** Qingwen Yang, Hina Zarrin, Tabea Hein, Burak Yildiz, Vivien Prex, Konstantinos Siontas, Tilman Borggrefe, Benedetto Daniele Giaimo, Tobias Friedrich, M Lienhard Schmitz, Thomas Wirth, Franz Oswald, Bernd Baumann

**Affiliations:** University Hospital Ulm, Department of Internal Medicine I, Albert-Einstein-Allee 23, 89081 Ulm, Germany; Department of Neurology, The Affiliated Suzhou Hospital of Nanjing Medical University, Suzhou 215002, China; University Hospital Ulm, Department of Internal Medicine I, Albert-Einstein-Allee 23, 89081 Ulm, Germany; University of Ulm, Institute for Physiological Chemistry, Albert-Einstein-Allee 11, 89081 Ulm, Germany; University Hospital Ulm, Department of Internal Medicine I, Albert-Einstein-Allee 23, 89081 Ulm, Germany; University of Ulm, Institute for Physiological Chemistry, Albert-Einstein-Allee 11, 89081 Ulm, Germany; University of Ulm, Institute for Physiological Chemistry, Albert-Einstein-Allee 11, 89081 Ulm, Germany; Justus-Liebig-University Giessen, Institute of Biochemistry, Friedrichstrasse 24, 35392 Giessen, Germany; Justus-Liebig-University Giessen, Institute of Biochemistry, Friedrichstrasse 24, 35392 Giessen, Germany; Justus-Liebig-University Giessen, Institute of Biochemistry, Friedrichstrasse 24, 35392 Giessen, Germany; Justus-Liebig-University Giessen, Institute of Biochemistry, Friedrichstrasse 24, 35392 Giessen, Germany; University of Ulm, Institute for Physiological Chemistry, Albert-Einstein-Allee 11, 89081 Ulm, Germany; University Hospital Ulm, Department of Internal Medicine I, Albert-Einstein-Allee 23, 89081 Ulm, Germany; University of Ulm, Institute for Physiological Chemistry, Albert-Einstein-Allee 11, 89081 Ulm, Germany

## Abstract

Brain aging is associated with chronic, low-grade inflammation known as inflammaging, which is largely driven by senescent glial cells secreting pro-inflammatory factors termed as senescence-associated secretory phenotype (SASP). In humans, the release of SASP factors in the brain is linked with an increased risk for the development of neurodegenerative disorders; thus, elucidating the underlying molecular mechanisms is essential for understanding the pathophysiology of brain aging. Both NF-κB and Notch signaling are known to regulate SASP expression; however, their mode of interaction in brain inflammaging is poorly understood. Here, we demonstrate that brain aging is broadly associated with NF-κB activation but Notch suppression using different transgenic mouse models. Astrocytic NF-κB activation in young mice was able to mimic premature SASP formation but repressed Notch signaling in parallel. Bioinformatic analyses further identified conserved crosstalk sites within SASP genes co-occupied by Notch/RBPJ and NF-κB transcription factors *in vivo*, allowing specific combinatorial regulation. Importantly, selective physical interactions enable that activated Notch can act as a negative modulator of NF-κB signaling at different molecular levels. Consistently, pharmacological activation of Notch in primary astrocytes significantly reduced NF-κB-driven SASP gene expression. Together, these findings identify Notch–NF-κB crosstalk as a potential therapeutic target to mitigate brain inflammaging.

## Graphical Abstract

**Graphical Abstract:**
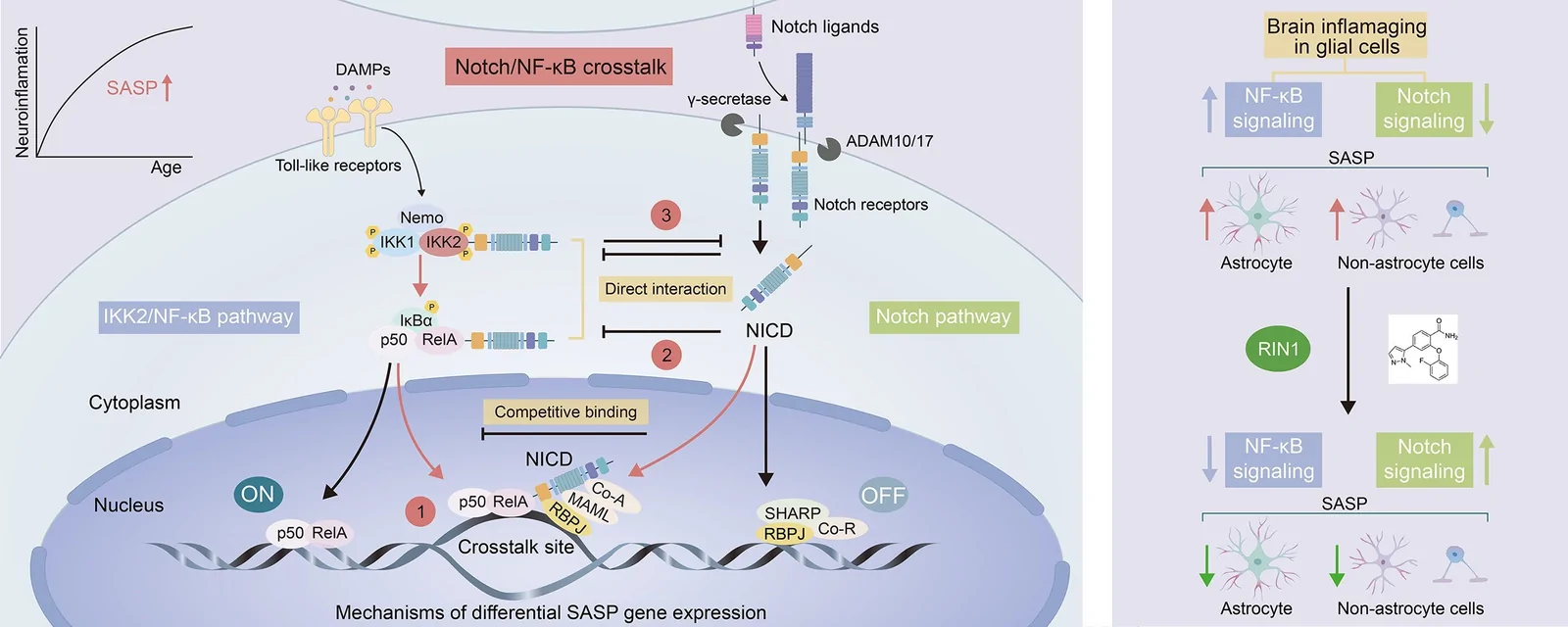

## Introduction

With physiological aging, brain homeostasis becomes progressively disturbed, leading to structural, biochemical, and neuroimmunological alterations that ultimately impair central nervous system (CNS) function. The accumulation of stress factors throughout life, including oxidative and genotoxic stress as well as self-derived molecules known as damage-associated molecular patterns (DAMPs), can promote a shift in the neuroimmune system toward a chronic, low-grade inflammatory state in the absence of overt infection, a process termed inflammaging [1]. Senescence of glial cells initiates the development of the senescence-associated secretory phenotype (SASP), which is recognized as a major driver of brain inflammaging. The release of diverse pro-inflammatory SASP factors create a detrimental neuroinflammatory environment, thereby promoting age-dependent synaptic dysfunction and cognitive decline [2] as well as predisposing for neurodegenerative disorders like Alzheimer’s and Parkinson’s disease [3]. Also, astrocytes and microglia change their reactive states, which can impair their homeostatic functions [4]. Thus, in the course of brain inflammaging the blood–brain barrier may get leaky facilitating the infiltration of peripheral leukocytes and thereby amplifying detrimental neuroinflammatory and neurodegenerative processes [5].

NF-κB is a well-known mediator of inflammatory and stress responses [6], whereas Notch signaling is a major intercellular communication pathway that regulates cellular differentiation and tissue homeostasis [7, 8]. Importantly, both pathways are critically involved in the inflammaging process and the development of cellular senescence, potentially triggering vicious cycle formation between inflammaged and senescent glial cells [9, 10, 11]. Notch and NF-κB pathways are proposed to interact on multiple levels, including shared DNA-binding site usage, reciprocal modulation of diverse signaling components, and mutual negative and positive feedback loops [12, 13, 14]. However, it remains unclear how these different crosstalk mechanisms orchestrate the multicellular inflammaging process in the CNS, and how brain function is generally affected.

Here, we deciphered the molecular basis of Notch/NF-κB crosstalk signaling in the aging brain and its role in regulating SASP gene expression. By integrating bioinformatic predictions with functional validation, we identified a subset of SASP genes co-regulated by both pathways and determined composite DNA-binding motifs containing recognition sites for both RBPJ and NF-κB subunits within their gene promoters. We also identified novel physical and functional interactions between key components of both pathways and further validated them *in vivo* using conditional transgenic mouse models and pharmaceutical modulation. Together, our findings reveal a multidimensional regulatory interplay between Notch and NF-κB signaling that orchestrates glial SASP expression during brain aging, thereby establishing a mechanistic framework for understanding and targeting brain inflammaging.

## Materials and methods

### Mice and animal procedures

Animal procedures were conducted in accordance with protocols approved by the Animal Facility of the University of Ulm and the Regierungspräsidium Tübingen, under the license O.110-9, in compliance with the German Animal Welfare Act. To investigate the crosstalk between Notch and NF-κB signaling in brain inflammaging, C57BL/6J, conditional gain- and loss-of-function (GOF and LOF) mouse models of IKK/NF-κB signaling in astrocytes (IKK2-CA^GFAP^ and IKK2-DN^GFAP^) were employed.

Mice conditionally expressing either a constitutively active IKK2 allele (IKK2-CA) or a dominant-negative acting allele (IKK2-DN) in astrocytes (IKK2-CA^GFAP^, IKK2-DN^GFAP^) were previously generated and reported [15, 16]. In brief, a transdominant (VSV-IKK2-DN; D145N) and a constitutively active version (CA-IKK2; IKK2-EE; S177E, S181E) of human IKK2 were cloned into the bidirectional tetracycline-dependent expression vector pBL5 to create the plasmids pBI5-VSV-IKK2-DN and pBL5-CA-IKK2. A bidirectional minigene was subsequently released by AatII-AseI; both fragments microinjected into the pronuclei of fertilized oocytes (NMRI outbred) and transplanted to pseudopregnant foster mothers [17]. To generate IKK2-CA^GFAP^ and IKK2-DN^GFAP^ double-transgenic mice, GFAP.tTA mice were crossed with (tetO)7.IKK2-CA) or (tetO)7.IKK2-DN) [18]. For transgene inactivation during complete development, doxycycline (0.5 g/l; MP Biomedicals) in 1% sucrose was given in the drinking water to the mothers during pregnancy from conception to weaning until the age of five weeks; then, transgene expression was induced by Dox withdrawal. IKK2-CA^GFAP^ and IKK2-DN^GFAP^ mice were bred on NMRI background, and single-transgenic and transgene-negative littermates were always used as controls. To monitor cell-type-specific NF-κB activation, we used an NF-κB-responsive reporter mouse line (kappa-EGFP) that uses EGFP as a read-out for *in vivo* NF-κB activity [19]. These mice were bred on pure 129/P2 Ola background.

Mice were maintained under specific pathogen-free conditions in the animal facility of Ulm University (TFZ, Ulm). Animals were kept in groups of 3–5 mice in a 12-h light–dark cycle and were given *ad libitum* access to food and water.

### Post-mortem sample preparation

For post-mortem analyses, animals were anesthetized with an IP injection of a mixture of ketamine (100 mg/kg) and xylazine (10 mg/kg) diluted in a 0.9% NaCl solution. For tissue fixation, transcardial perfusion was performed, first with 25 ml of ice-cold PBS to allow blood removal, followed by 50 ml of ice-cold 4% Paraformaldehyde (PFA) in PBS. Brains were extracted and further postfixed in 4% PFA for 24 h, transferred to a solution of 30% saccharose in PBS at 4°C overnight for cryoprotection, and stored at 4°C until use.

### Flow cytometry

Living cells were transferred into a fixed volume and numbers were determined using Neubauer improved counting chamber. Staining for flow cytometric phenotyping was performed in PBS + 5% FCS + 0.05% Sodium Azide (FACS staining buffer). Dissociation of brains was performed using the Multi Tissue Dissociation Kit 1 (Miltenyi Biotec, Germany), according to the manufacturer’s protocol, Fc receptors were blocked for 15 min (FcR Blocking Reagent mouse, 130-092-575, Miltenyi Biotec, Bergisch Gladbach, Germany), and cells were incubated with the respective conjugated antibodies for 20 min. Flow cytometry was performed with the BD FACSCanto II, and analysis was performed using FlowJo^™^ 10.7.1 (FlowJo LLC, BD). Living cells were gated by a combination of forward/side scatter and DAPI. The antibodies used for flow cytometry analysis were listed in Supplementary Table S11.

### Primary cell isolation

Primary astrocytes and microglia from adult mice were isolated using the Adult Brain Dissociation Kit, gentleMACS Octo Dissociator with Heaters and MS columns in a magnetic field (Miltenyi Biotec, Bergisch-Gladbach, Germany), according to the manufacturer’s protocol. In brief, brains were dissected and enzymatically digested, and the debris was removed. Four-to-six hemispheres were pooled per sample. The remaining single cell suspension was first Fc-blocked and labeled with anti-ACSA-2 microbeads (130-097-679, Miltenyi Biotec), washed, and applied to an MS column. Purified ACSA-2 positive astrocytes were pelleted and snap-frozen in liquid nitrogen. The flowthrough was subsequently labeled with anti-CD11b microbeads (130-049 601, Miltenyi Biotec), washed, and purified using an MS column. The cell pellet containing CD11b positive cells was snap-frozen in liquid nitrogen. ACSA-2 and CD11b isolates were stored at −80°C until further analysis. For primary astrocyte cell culture, purified ACSA-2^+^ astrocytes were plated on Poly-D-Lysin coated plates using serum-free astrocyte medium. After 24 h, cells were left untreated or treated with RIN1 (10 µM) and/or a cocktail of LPS (10 µg/ml)/TNF-α (100 pg/ml)/Poly I:C (2 µg/ml) for 16 h to stimulate the IKK/NF-κB signaling pathway. Subsequently, cells were harvested and RNA was isolated.

### RNA extraction, cDNA synthesis, and qPCR

RNA of cortical tissues and primary cells were isolated using a PeqGOLD^®^ TriFast^™^ Kit (peQlab, Darmstadt, Germany) and Qiagen RNeasy Kits (Qiagen), according to the manufacturer’s instructions. For cDNA synthesis, the Transcriptor High fidelity cDNA synthesis kit (Roche, Penzberg, Germany) was used according to the manufacturer’s instructions but with only 0.5 µl instead of 1 µl reverse transcriptase mix and oligo-dT primers alone. To perform qRT-PCR, we used the LightCycler^®^ 480 Instrument (Roche) according to the manufacturer’s instructions with intron spanning primers and mono-color hydrolysis probes designed by Roche’s Universal Probe Library UPL assay design center. The following housekeeping genes were used as reference genes: hypoxanthine-guanine phosphoribosyl transferase (*Hprt1*) and beta glucuronidase (*Gusb*). The housekeeping genes used for normalization are specified in the figure legends. Primer sequences and UPLs are available upon request.

### RNA sequencing of primary cells

RNA sequencing was conducted by Novogene (London, UK). In summary, mRNA was purified from total RNA using poly-T oligo-attached magnetic beads. After the fragmentation step, random hexamer primers were used for first-strand cDNA synthesis, which was followed by the second-strand cDNA synthesis using dUTP for the directional library. The library was afterward checked by Qubit and real-time PCR for quantification, for size distribution detection, a bioanalyzer was used. Index-coded samples were clustered according to the manufacturer’s instructions. After cluster generation, the library preparations were subsequently sequenced on an Illumina platform. Paired-end reads were generated, and raw data (raw reads) of FASTQ format were processed through fastq. Reads containing adapter and poly-N sequences, as well as reads with low quality were removed in this step to obtain clean data (clean reads). Q20, Q30, and GC content of the clean data were calculated, and for all downstream analyses, clean data of high quality were used. GRCm38 reference genome as well as gene model annotation files were directly downloaded from the genome website browser (NCBI/UCSC/Ensembl). Paired-end clean reads were aligned to the reference genome using the Spliced Transcript Alignment to a Reference (STAR) software. This software is based on a previously undescribed RNA-seq alignment algorithm that uses sequential maximum mappable seed search in uncompressed suffix arrays, which is followed by seed clustering and stitching procedure. Compared to other RNA sequencing aligners, STAR exhibits better alignment precision and sensitivity in both experimental and simulated data. FeatureCounts was used to count the read numbers mapped of each gene; reads per kilobase of exon model per Million mapped reads (RPKM) were calculated based on the gene length and reads count mapped to this gene. With RPKM, the effects of sequencing depth and gene length for the read count are considered at the same time. Currently, it is the most commonly used method for estimation of gene expression levels [20]. DEGS were calculated by DESeq2, heatmaps of DEGS were generated using R (Version 3.0.3) ggplot2 package, pheatmap package. For Gene Set Enrichment Analysis, GSEA 4.3.3 by UC San Diego and Broad Institute ( https://www.gsea-msigdb.org/gsea/msigdb) was utilized [21, 22].

### ChIP-Seq

Cells were crosslinked in 0.37% Formaldehyde (FMA) for 10 min at room temperature. The FMA reaction was blocked by adding 1/8 volume of 1 M glycine pH 7.5 and incubation for 5 min at room temperature. Fixed cells were washed twice with PBS. ChIP was essentially done as previously described [23] using an anti-RBPJ antibody (Cell Signaling Technology, 5313S). Libraries were prepared using the Diagenode MicroPlex Library Preparation Kit v3 (Diagenode C05010001) following manufacturer’s instructions with few modifications. Libraries were purified with Agencourt AMPure XP Beads (Beckman Coulter, #A63881), quantified, and analyzed on an Agilent Tapestation device. Finally, sequencing was done on a NovaSeq6000 device at Novogene UK.

### ChIP-Seq analysis

Raw sequencing reads were adapter and quality trimmed using Trim Galore (https://github.com/FelixKrueger/TrimGalore). Trimmed reads were aligned against the human reference genome (hg19) using HISAT2 [24] with “–no-spliced-alignment” parameter and stored as BAM files. BAM files were filtered for PCR duplicates using Picard tools (https://broadinstitute.github.io/picard/). Peak calling was performed using Peakranger [25] with the corresponding input. Peaks were annotated to their corresponding gene using ChIPseeker [26]. Motif identification was performed using MEME-suite [27].

### *In silico* analysis of transcription factor binding sites

Chromatin accessibility and transcription factor binding patterns were analyzed using ChIP-seq datasets targeting RBPJ and ATAC-seq datasets from *Mus musculus* (mm10) and *Homo sapiens* (hg38), with a focus on the transcription factors RBPJ, RelA, and NF-κB1 (p50). These datasets were visualized using the Integrative Genomics Viewer (IGV, v2.16.2) to identify binding peaks and assess chromatin accessibility, with H3K27Ac histone modification serving as a marker for active transcription. Promoter regions were defined as the first exon and intron plus up to 15 kb upstream of the transcription start site. Regions of interest were those with double occupancy (bound by both RBPJ and RelA/NF-κB1). Precise coordinates for these regions were extracted from IGV and cross-referenced in the Ensembl Genome Browser to obtain detailed information about chromosome regions, genes, and regulatory annotations. Sequences of interest were exported in FASTA format and analyzed for transcription factor binding motifs using the PROMO database, supplemented with manual annotation of RBPJ-binding sequences (e.g. TGGGA/TCCCA).

### Plasmid construction and preparation

The 12×RBP and 3×κB reporter plasmids are well-established constructs that specifically report Notch and NF-κB pathway activity, respectively, and have been described previously. Reporter plasmids containing 3×CT-sites derived from promoter of representative SASP genes (*Hes1, Il6, Ifnb1, Lcn2, Cdr1, Igk*, and *Tnip1*) were generated by sequential digestion and ligation (Supplementary Table S8). Plasmid backbones (pGL3-12×RBP) and synthesized insert fragments in pUC57-BsaI-Free (BioCat) were digested with HindIII and NsiI, followed by gel purification using the QIAquick Gel Extraction Kit (Qiagen).

Purified fragments were ligated with T4 DNA ligase at room temperature for 2 h. Ligated plasmids were transformed into *Escherichia coli* XL1-Blue competent cells, and minipreps were performed using the Wizard^®^Plus SV kit. For large-scale plasmid preparation, EndoFree^®^ Plasmid Maxi kits were employed, according to manufacturer protocols. Further details of plasmids (Supplementary Tables S9 and S10) can be provided upon request.

### Cell culture and transient transfection

Kasumi-1 cells (acute myeloid leukemia, DSMZ, ACC 220) were grown in Roswell Park Memorial Institute (RPMI-1640, Gibco 61870-010) medium 1640 (1×) with addition of 20% fetal calf serum (FCS, #S0115, Biochrom) and 1% penicillin/streptomycin (Thermo Fisher Scientific). HEK293 (ATCC: CRL-1573) and HeLa (ATCC: CCL2), and HeLA-RelA^KO^ [28] derived cell lines were maintained in DMEM supplemented with 10% fetal calf serum and 1% penicillin/streptomycin at 37°C in a humidified atmosphere with 5% CO₂. C8-D1A (C57BL/6 cerebral cortex astrocytes, Cytion 300 316) were cultures in DMEM (Gibco 41 966 029) with 10% fetal calf serum and 1% antibiotic-antimycotic (Gibco 15240062). Cells were passaged at approximately 80% confluency using trypsinization. For HeLa cells, an extended incubation of 3 min at 37°C was used, whereas C8-D1A cells were incubated for 5 min at room temperature to facilitate detachment. Detached cells were resuspended in prewarmed medium. Cells were seeded into appropriate culture vessels depending on the experimental setup. Transient transfections were performed using Lipofectamine^™^ 2000 (Thermo Fisher Scientific) for HeLa, HeLa-RelA^KO^, and C8-D1A cells, while the ProFection^®^ Calcium Phosphate System (Promega) was used for HEK293 cells, following the manufacturers’ protocols.

### Luciferase reporter assay

HeLa and HeLa-RelA^KO^ cells were seeded in 48-well plates at a density of 2.25 × 10^4^ cells per well, while C8-D1A cells were seeded at a density of 2 × 10^4^ cells per well to achieve 70%–90% confluency after 24 h. Lipofectamine^™^ 2000 (Thermo Fisher Scientific) and DNA were separately diluted in Opti-MEM (Thermo Fisher Scientific), combined, and incubated for 5 min at room temperature before addition to cells. After 24 h incubation at 37°C, cells were lysed in 100 μl of 1× Luciferase Cell Culture Lysis Reagent (Promega, Cat. no. E1531). Lysates were incubated for 30 min at room temperature with shaking and centrifuged at 20 817 × *g* for 4 min at 4°C. For luciferase activity measurement, 10 μl of supernatant were mixed with 50 μl of luciferase substrate (Promega, Cat. no. E1501) in 96-well plates, and luminescence was measured using a Centro LB 963 luminometer (Berthold) with a 2 s delay and 5 s integration.

For luciferase assay in tissues, luciferase activity was measured in protein extracts from various organs (heart, thymus, spleen, liver, kidney, and pancreas) and brain regions (medulla, cerebellum, and cortex) to validate the functionality and tissue specificity of transgene expression as described previously [18, 29]. Protein extracts in luciferin-containing buffer were analyzed in a luminometer (Lumat), and luminescent readings (in RLU) were normalized to the corresponding protein concentrations.

For *in vivo* bioluminescence imaging, mice were injected i.p. with D-luciferin and anaesthesized for *in vivo* imaging of luciferase bioluminescence in an IVIS 200 System (Calipher).

### Immunofluorescence and fluorescence microscopy

HeLa cells and HeLa-RelA^KO^ cells were seeded at a density of 4.0 × 10^4^ cells per well onto fibronectin-coated two-well chambered coverslips (fibronectin 1:10 in PBS, incubated 30 min at 37°C). Cells were transfected with plasmids using Lipofectamine^™^ 2000 as described above and incubated for 24 h at 37°C. Cells were fixed with 4% paraformaldehyde (Merck KGaA) for 15 min at room temperature, followed by quenching with 50 mM ammonium chloride (Sigma–Aldrich) for 10 min. For immunofluorescence assays, cells were stained with an anti-RelA antibody (rabbit anti-p65 polyclonal, #51-0500, Thermo Fisher Scientific/Invitrogen), followed by incubation with an anti-rabbit secondary antibody (alexa Fluor^®^ 488 conjugate, #A27034, Thermo Fisher Scientific/Invitrogen). After washing, actin staining was performed using Alexa Fluor^®^ 568 conjugated phalloidin (#A12380; Thermo Fisher Scientific). Nuclei were stained with DAPI (1:1000 in PBS, Sigma–Aldrich) for 30 min. Samples were mounted using Fluoromount-G (Southern Biotech) and imaged using an Olympus IX81 fluorescence microscope equipped with the following filter sets: DAPI (excitation: 360/40, emission: 457/50), EGFP/Alexa flour^™^-488 (excitation: 470/40, emission: 525/50), YFP (excitation: 500/20, emission: 535/30), and Cy3 (excitation: 545/25, emission: 605/70). Images were acquired using Simple PCI software (Hamamatsu), and analyzed with ImageJ.

### Protein extraction from cells and mouse brain

HEK293 cells were harvested 24 h post-transfection for protein extraction. Following removal of the culture medium, cells were washed with PBS and detached using pre-warmed trypsin at 37°C for 4 min. After centrifugation at 106 × *g* (Eppendorf Refrigerated Centrifuge 5417R) for 7 min at 4°C, the cell pellet was washed with ice-cold PBS and centrifuged again at 153 × *g* for 6 min. The resulting pellet was lysed in CHAPS buffer [10 mM CHAPS (Sigma–Aldrich), 50 mM Tris/HCl pH 7.8, 150 mM NaCl] supplemented with protease inhibitors [5 mM NaF, 0.5 mM PMSF (Sigma–Aldrich), 40 μl/ml Complete^™^ protease inhibitor cocktail (Roche)] for 1 h to facilitate complete cell lysis.

For brain protein extraction, mouse brains were rapidly dissected and placed in ice-cold PBS. Non-cortical regions (olfactory bulbs, cerebellum, and brainstem) were removed, and the remaining cerebrum was snap-frozen in liquid nitrogen and stored at –80°C. Frozen tissue was pulverized under liquid nitrogen, and RIPA buffer [150 mM NaCl, 50 mM Tris/HCl, 0.93% (v/v) Triton X-100, 1% (w/v) sodium deoxycholate (DOC, Sigma–Aldrich), and 0.1% (w/v) Sodium dodecyl sulfate (SDS, Carl Roth)] supplemented with PhosStop EasyPack phosphatase inhibitor (Roche) was added at a 3:1 buffer-to-tissue volume ratio. Lysates were incubated on ice for 30 min with intermittent vortexing, followed by centrifugation at 20 817 × *g* for 10 min at 4°C. The supernatant was collected and stored at –80°C.

### Co-immunoprecipitation

Co-immunoprecipitation (Co-IP) assays were performed to analyze protein–protein interactions using both direct and indirect approaches. For direct Co-IP of Flag-tagged proteins, 50 μl of anti-Flag M2 affinity gel (Sigma–Aldrich, Cat. no. A2220) was prepared per sample and washed five times with 0.5% BSA in PBS, followed by one wash with CHAPS buffer (425 × *g*, 1 min, 4°C). The beads were then resuspended in 2/3 of the original volume using CHAPS buffer. Subsequently, 500 μg of lysate in CHAPS buffer (supplemented with protease inhibitors, as described above) was incubated with the beads overnight at 4°C on the overhead rotator. For indirect Co-IP of endogenous or non-tagged proteins, lysates were first incubated overnight at 4°C with either anti-GFP (Merck KGaA, Cat. no. 11814460001), anti-HA (Merck KGaA, Cat. no. 11867423001), or anti-IKKβ (Cell Signaling Technology, Cat. no. 8943) antibodies. Immune complexes were subsequently captured using 50 μl of Protein G Sepharose 4 Fast Flow (Cytiva, Cat. no. 17061801) or Protein A Agarose Beads (Cell Signaling Technology, Cat. no. 9863) for 2 h at 4°C. Appropriate beads were pre-washed three times with cold PBS (425 × *g*, 1 min, 4°C) and once with CHAPS buffer to minimize non-specific binding. All bead–protein complexes were washed six times with CHAPS buffer containing Complete^™^ protease inhibitor cocktail (40 μl/ml, Roche) and subsequently denatured at 95°C for 5 min in 45 μl of Laemmli buffer.

### Co-stimulation in C8-D1A cells

C8-D1A cells were seeded in 10-cm dishes at a density of 1 × 10^6^ cells per dish to achieve 70%–80% confluency after 24 h. Cells were treated with the Notch agonist RIN1 (20 µM, 24 h), TNF-α (50 ng/ml) either for 30 min or 24 h, or a combination of both, with Dimethylsulfoxide (DMSO) as a negative control. Cells were harvested 24 h post-stimulation for protein extraction. Following removal of the culture medium, cells were washed with PBS and detached using cell scraper. After centrifugation at 300 × *g* (Eppendorf Refrigerated Centrifuge 5417R) for 3 min at 4 °C, the cell pellet was washed with ice-cold PBS and centrifuged again at 300 × *g* for 5 min. The resulting pellet was lysed in CHAPS buffer [10 mM CHAPS (Sigma–Aldrich), 50 mM Tris/HCl pH 7.8, 150 mM NaCl] supplemented with protease inhibitors [5 mM NaF, 0.5 mM PMSF (Sigma–Aldrich), 40 μl/ml Complete^™^ protease inhibitor cocktail (Roche), 100 μl/ml PhosStop EasyPack phosphatase inhibitor (Roche)] for 1 h to facilitate complete cell lysis, followed by centrifugation at 20 817 × *g* for 30 min at 4 °C. The supernatant was collected and stored at −80°C.

### Western blotting

SDS–polyacrylamide gel electrophoresis (SDS–PAGE) was used for the separation of proteins based on their molecular weight. A 10% resolving gel was used for proteins below 55 kDa, and a 7.5% gel was used for higher molecular weight proteins. After electrophoresis, proteins were transferred onto an Immobilon^®^-P PVDF Membrane (Merck, Cat. no. IPVH00010) using a three-buffer system composed of buffer 1 [0.3 M Tris–HCl, pH 10.4, 20% (v/v) methanol], buffer 2 [0.03 M Tris–HCl, pH 10.4, 20% (v/v) methanol], and buffer 3 [0.04 M β-aminocaproic acid, pH 9.4, 20% (v/v) methanol]. Transfers were carried out in a semi-dry blotting at 250 mA for 90 min at room temperature. Primary and secondary antibodies used in this study are listed in Supplementary Table S11. For signal detection, membrane was incubated with either Amersham ECL Western Blotting Detection Reagent (Cytiva, Cat. no. RPN2106) or SuperSignal West Femto Maximum Sensitivity Substrate (Thermo Fisher Scientific, Cat. no. 34 095), followed by chemiluminescent imaging.

### Statistical analysis

Quantitative data are presented as mean ± standard deviation (SD). Statistical analyses were performed using GraphPad Prism 9. Comparisons between 2 groups were analyzed unpaired two-tailed Student’s *t*-test or Mann–Whitney *U* test, as appropriate. For comparisons involving three or more groups, when variances were equal, one-way ANOVA followed by Tukey’s multiple comparisons test was performed. In cases of unequal variances, Brown–Forsythe or Welch’s ANOVA was employed, followed by Dunnett’s T3 post-hoc test. Statistical significance was defined as *P*-value < 0.05. Statistical details for each analysis and the number of independent experiments can be found in the figure legends.

## Results

### Age-related NF-κB activation in glial cells coincides with a decline in Notch target gene expression

To monitor age-dependent activity changes in IKK/NF-κB signaling in the CNS, we analyzed EGFP-expression in the brain from young (3 months) and aged (24 months) NF-κB reporter gene mice (kappa-EGFP) [30]. Immunohistochemical EGFP-staining revealed a marked increase in the number of cells exhibiting NF-κB activity in aged brains (Fig. 1a). To further characterize the brain cells with NF-κB activation, we performed flow cytometric analysis using MACS-dissociated whole-brain tissue. The overall frequency of EGFP-positive brain cells was approximately doubled from young to aged mice (Fig. 1b, upper left). Interestingly, the number of both ACSA-2⁺astrocytes (Fig. 1b, upper, right) and mature oligodendrocytes (O4⁺) (Fig. 1b, lower, left) showing activated NF-κB signaling was profoundly upregulated from young to old mice, whereas microglia/myeloid cells (CD11b^+^/CD45^+^) with activated NF-κB were already elevated at 3 months of age, which nearly doubled by 24 months (Fig. 1b, lower right). As a consequence, increased transcription of NF-κB target/SASP genes like *C3, Lyz2, C4b* (Fig. 1c), *Mmp12, Tnf*, and *Bcl3* (Supplementary Fig. S1a) was determined in the cortex of old mice. Also, *Cdkn2a*, a well-known marker of senescent cells and aged tissue, was found upregulated in aged brains (Supplementary Fig. S1b).

**Figure 1.**
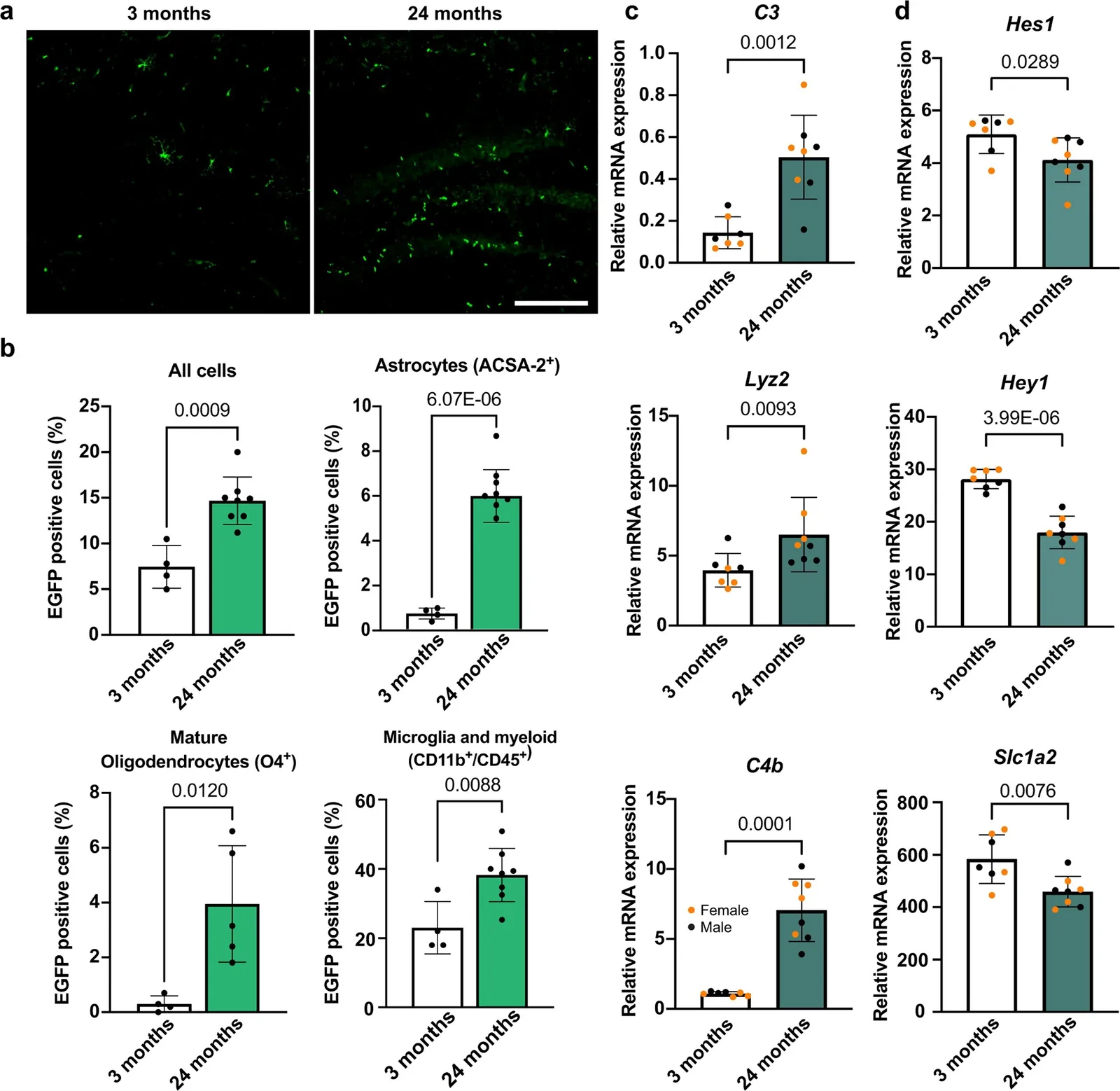
Aging induces NF-κB activation and suppresses Notch signaling in the mouse brain. (**a**) Immunohistochemical staining of brain sections from NF-κB reporter mice (κB-EGFP) revealed a marked increase in the number of cells exhibiting NF-κB activity in aged (24 months) compared with young (3 months) mice; scale bar: 100 μm. (**b**) Flow cytometric analysis of MACS-dissociated whole-brain tissue from NF-κB reporter mice, quantifying the total number of EGFP-positive cells as well as EGFP-positive astrocytes (ACSA-2⁺), mature oligodendrocytes (O4⁺), and microglia/myeloid cells (CD11b⁺/CD45⁺) in young (3 months, *n* = 4) and aged (24 months, *n* = 8) mice. (**c, d**) RT-qPCR analysis of NF-κB target/SASP genes (*C3, Lyz2*, and *C4b*) and Notch target genes (*Hes1, Hey1*, and *Slc1a2*) in the cortical tissue from young (3 months, *n* = 7) and aged (24 months, *n* = 8) mice (C57BL/6J), revealing increased NF-κB-driven SASP gene expression and concomitant with reduced transcription of Notch target genes in aged brains. Data were normalized to *Gusb*. Data are shown as mean ± SD. Statistical analysis: unpaired two-tailed Student’s *t*-test or Mann–Whitney test.

To test the activity changes in Notch signaling, we analyzed the expression of canonical Notch target genes in young and aged mice. Notably, mRNA levels of *Hes1, Hey1*, and *Slc1a2* (Fig. 1d), and *Hey2, Dtx1, Bcl2*, and *Slc1a3* (Supplementary Fig. S1c) were significantly decreased in aged mice, while expression of the Notch pathway components *Rbpj, Notch1, Notch2*, and *Notch4* was hardly affected (Supplementary Fig. S1d). In summary, we could identify NF-κB activation coming along with a decline in Notch signaling during brain aging in wild-type mice.

### NF-κB activation in astrocytes is sufficient to initiate premature SASP development accompanied with Notch suppression

Having confirmed prominent, age-dependent NF-κB activation in glial cells, we focused on astrocytes, which exhibited a marked upregulation and also represent the major brain-resident glial population critically regulating CNS homeostasis and maintenance. For this, we employed a gain-of-function mouse model for NF-κB called GFAP.tTA/tetO.IKK2-CA (IKK2-CA^GFAP^) [18, 31] to uncover the consequences of conditional IKK/NF-κB activation in astrocytes at young age. Bioluminescence measurements confirmed brain-restricted transgene activity in IKK2-CA^GFAP^ mice, showing luciferase activity in the cerebellum, medulla, and cortex but not in peripheral organs (Supplementary Fig. 2a,b). Previous work already demonstrated a global neuroinflammatory phenotype exhibiting prominent astro- and microgliosis in IKK2-CA^GFAP^ animals at the age of one year [32]. This phenotype strongly suggests an IKK2/NF-κB driven SASP development, a process usually emerging only in the brain of elderly mice.

To further characterize this premature brain aging phenotype in IKK2-CA^GFAP^ mice, we performed gene expression analysis of selected SASP genes using cortical tissue from 3-month-old IKK2-CA^GFAP^ mice and compared them with young (3-month-old) and aged (24-month-old) control littermates. In control mice, typical representatives of SASP genes (e.g. *Il1b, Cst7*, and *Mmp12*) were upregulated in an age-dependent manner (Fig. 2a). Strikingly, IKK2-CA^GFAP^ animals exhibited an elevated expression of these age-regulated genes already at 3 months of age. Taking a closer look at additional immune-related genes (*Lyz2, Cd68*, and *Ly9*) and tissue remodeling-associated gene *Tgm1*, we again identified a similar pattern with significantly increased expression levels in both 24-month-old controls and young IKK2-CA^GFAP^ mice (Fig. 2a).

**Figure 2.**
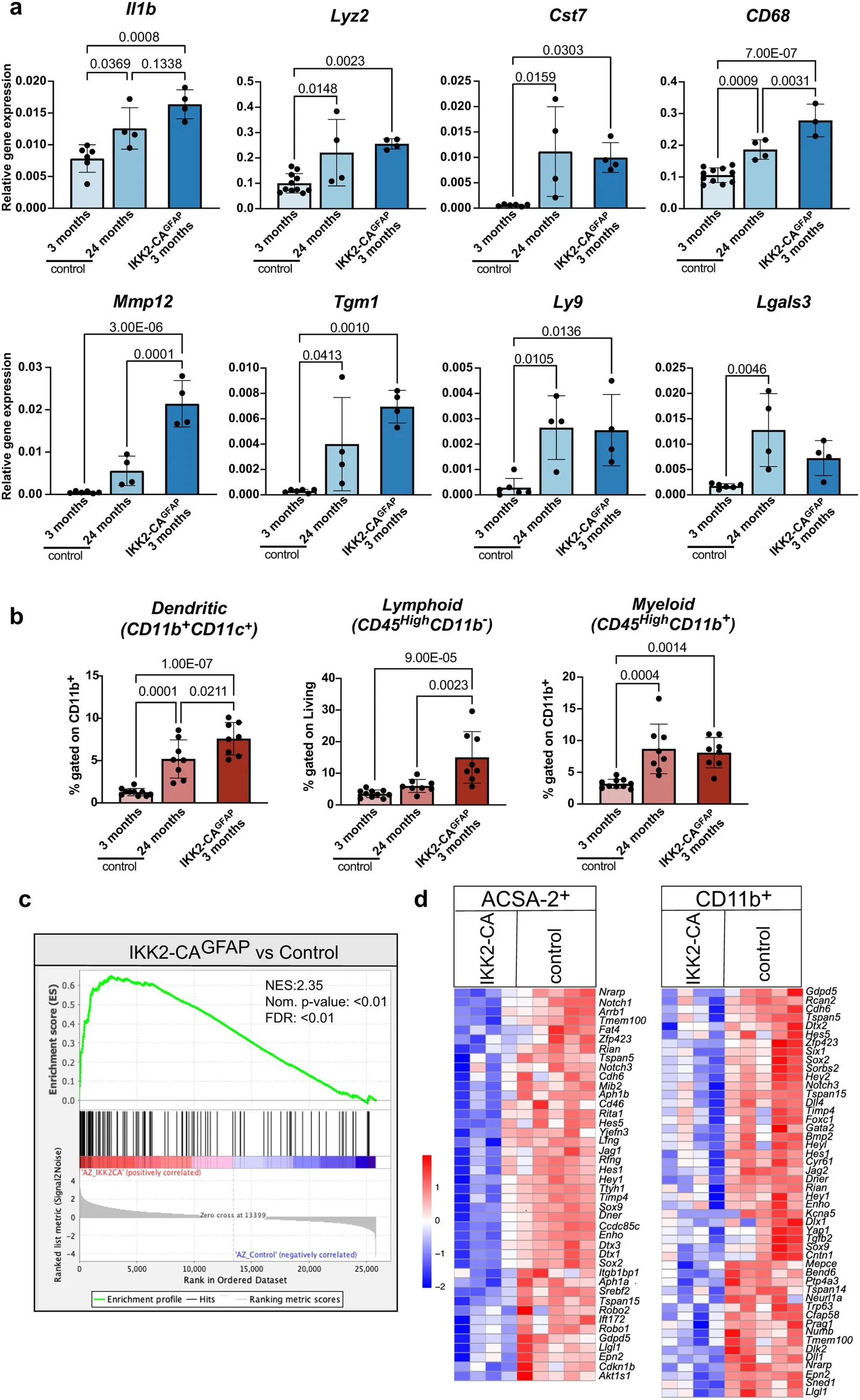
Astrocytic NF-κB activation drives premature brain inflammaging, SASP gene induction, and Notch pathway suppression in astrocytes and microglia. (**a**) qRT-PCR analysis of representative SASP genes (*Il1b, Lyz2, Cst7, CD68, Mmp12, Tgm1, Ly9*, and *Lgals3*) in cortical tissue from young (3 months) and aged (24 months) wild-type mice, and young (3 months) IKK2-CA^GFAP^ mice (*n* = 4–6/group). Young IKK2-CA^GFAP^ mice show premature upregulation of SASP genes comparable to aged controls. Data were normalized to *Hprt*. (**b**) Flow cytometric analysis of dissociated brains of IKK2-CA^GFAP^ mice showing frequencies of CD45^bright^/CD11b⁺ myeloid cells, CD45^high^/CD11b⁻ lymphoid cells, and CD11b⁺CD11c⁺ cells in young and aged controls as well as young IKK2-CA^GFAP^ animals. (**c**) Gene set enrichment analysis of MACS-purified astrocytes (ACSA-2^+^) from young (18–22 weeks) IKK2-CA^GFAP^ mice (*n* = 4) and control littermates (*n* = 5) showing significant enrichment of SASP-related genes (FDR < 0.01, NES = 2.37, nominal *P*-value < 0.01) (MM16098). (**d**) Heatmap of RNA-seq data showing downregulation of the Notch gene signature (GO:0 007 219) in astrocytes (left, ACSA-2^+^) and microglia (right, CD11b^+^) from IKK2-CA^GFAP^ mice (*n* = 4) compared with controls (*n* = 5). Data shown as mean ± SD. Statistical analysis: one-way ANOVA with Tukey’s multiple comparisons test. Analysis of RNA sequencing by DESeq2 (two-sided Wald’s test), |log_2_FoldChange| > 1, padj-value < 0.05. FDR correction of *P*-values (padj) by multiple comparisons test. Over- or underrepresentation of gene sets (GSEA analysis) identified by a gene set-based permutation test and subsequent multiple hypothesis testing.

To gain a more comprehensive insight into the gene expression changes of specific cell populations, a MACS purification of astrocytes (ACSA-2^+^) and myeloid/microglia (CD11b^+^) was conducted with brain samples of young adult IKK2-CA^GFAP^ animals and control littermates (Supplementary Fig. S3a). Principal component analysis (PCA) revealed prominent changes in the transcriptome of both IKK2-activated astrocytes and microglia/myeloid cells in comparison to the corresponding control population (Supplementary Fig. S3b), leading to the formation of distinct clusters. Differential gene expression analysis identified 2309 upregulated and 674 downregulated genes in astrocytes of IKK2-CA^GFAP^ mice as compared to control littermates (Supplementary Fig. S3c). Several of the top upregulated genes such as *Ccl22, C3*, and *Ccl17* are associated with inflammation and immune functions, which is also reflected in the enriched pathways obtained from the GO term database (Supplementary Fig. S3d). Categorizing the results listed in the enrichment charts; significantly upregulated processes mainly include cytokine and chemokine signaling, lymphocyte activation and chemotaxis, innate immune defense, and interferon-related pathways such as the terms “MHC protein complex” (Supplementary Fig. S3d) and “Interferon type II production” (Supplementary Fig. S3e). However, GO terms associated with metabolic and neuronal functions such as learning, synapse, and channel expression were significantly downregulated (Supplementary Table S1). In line with this, astrocytes developed a significant SASP gene signature (Fig. 2c). Interestingly, this prominent SASP gene expression profile is accompanied by a downregulation of components in the Notch pathway signature (GO:0007219, GO:0045747) both in astrocytes and CD11b^+^ myeloid cells (Fig. 2d, Supplementary Fig. S3f, g, h, Supplementary Fig. S4d, e), with a more pronounced effect in the latter population. These data support the idea that IKK2-CA^GFAP^ mice phenocopy transcriptomic traits of the aged brain already in early life.

CD11b^+^ myeloid cells of IKK2-CA^GFAP^ animals, similar to astrocytes, also revealed a profound enrichment in inflammatory gene expression signatures (Supplementary Fig. S3d, Supplementary Fig. S4a, b) including the interferon type II pathway (Supplementary Fig. S4b). In addition, CD11b^+^ cells show an expression pattern similar to the DAM (disease-associated microglia) phenotype (Supplementary Fig. S4c) [33, 34], a signature observed in various models of neurodegeneration and aging [35, 36]. This indicates that paracrine signaling of IKK/NF-κB activated astrocytes is able to force the polarization of microglia to a DAM-like state. Related to this finding, CD11b^+^ isolates of IKK2-CA^GFAP^ animals mount a gene expression signature previously described for aged myeloid/microglial cell (Supplementary Fig. S4f).

Taken together, our data strongly suggest that astrocytic activation of the IKK/NF-κB signaling is sufficient to induce a brain inflammaging-like phenotype, characterized by suppression of the Notch pathway, premature SASP gene expression and the development of an age-related neuroimmune phenotype.

### Aging-relevant genes harbor specific composite RBPJ/NF-κB binding motifs

To gain mechanistic insight into the potential crosstalk between Notch and NF-κB signaling in the regulation of SASP gene expression, we performed an unbiased ChIP-seq analysis (GSE314541) of RBPJ-bound genomic regions using a human myeloid cell line (Kasumi-1). Among 266 identified RBPJ-bound regions, 88 were identified either as RBPJ/NF-κB-CT-sites (cross-talk sites), which could occur with or without additional NF-κB-sites, or as RBPJ sites located adjacent to independent NF-κB sites on the same read (Fig. 3a, b, and Supplementary Table S2). Interestingly, pathway analysis using the PANTHER classification system (https://pantherdb.org/ ) revealed that genes harboring such RBPJ/NF-κB-CT-sites together with one or more additional NF-κB sites within their promoter region were strongly associated with inflammatory and aging-related pathways (Fig. 3c) further suggesting a relevant interaction of both pathways in aging.

**Figure 3.**
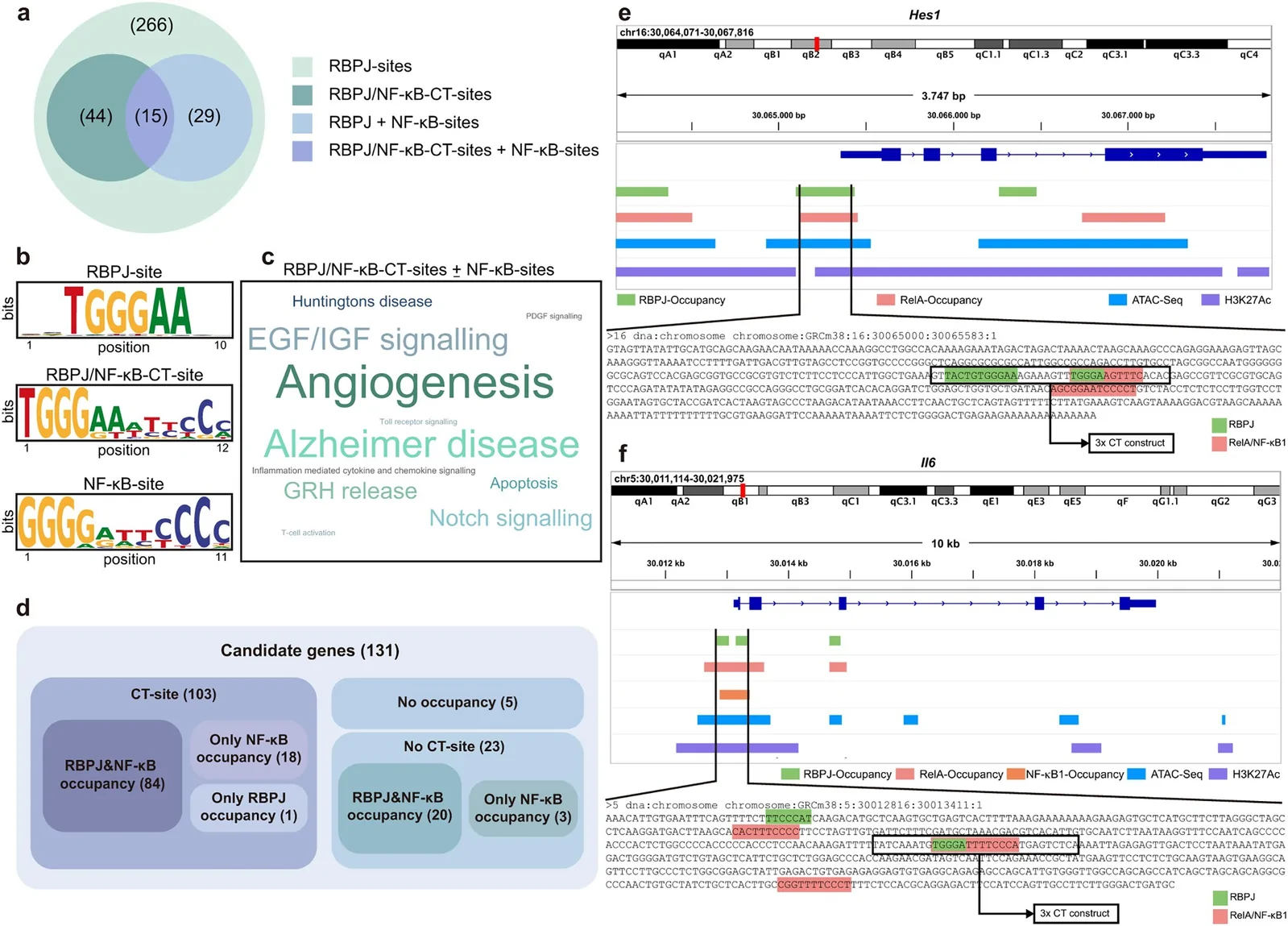
Identification of RBPJ/NF-κB CT-sites in aging-relevant genes. (**a, b**) ChIP-seq analysis of RBPJ-bound genomic regions in a human myeloid cell line identified 266 RBPJ peaks located at active promoter regions. Within these RBPJ bound peaks, we identified RBPJ/NF-κB-CT-sites (59), one or more NF-κB sites next to an RBPJ-site [29] or together with the RBPJ/NF-κB-(CT) sites [15]. (**c**) Pathway analysis of RBPJ bound genes with identified NF-κB sites and/or RBPJ/NF-κB-CT-sites was performed. Font size reflects the number of genes within a given pathway. (**d**) Candidate genes linked to brain aging, SASP, and Notch/NF-κB signaling were categorized according to the presence of RBPJ/NF-κB-CT sites and the occupancy by RBPJ and/or NF-κB. A total of 131 candidate genes were analyzed. (**e, f**) CT-sites in the promoter regions of *Hes1* (e) and *Il6* (f) are depicted using genome browser views (IGV interface, Mus musculus genome assembly mm10). The top panels display the genomic positions and sizes of genes (dark blue). Chromosomal positions are indicated along the top scale. The middle panels show ChIP-seq signal tracks for transcription factors RBPJ (green), RelA (red), and NF-κB1 (orange), along with chromatin accessibility (ATAC-seq, blue), and active histone marks (H3K27Ac, purple). Regions of interest were defined as promoter-associated sites exhibiting double occupancy by RBPJ and RelA/NF-κB1. The bottom panels show the nucleotide sequences of these regions within the *Hes1* and *Il6* promoters. RBPJ-binding sites (green), RelA/NF-κB1 binding sites (red), and CT-sites (overlapping regions) are highlighted. Sequences enclosed in black rectangles represent CT-sites that were used for reporter construct generation.

To further pursue this hypothesis, we conducted a comprehensive *in silico* screening for CT-site DNA motifs capable of binding both RBPJ and NF-κB within age-related genes. We compiled a list of “brain aging–related candidate genes,” defined by the following criteria: (i) genes known to be transcriptionally active in glial cells, (ii) candidates prioritized in our ChIP-seq analyses, (iii) genes related to SASP and brain aging described in public databases, RNA-seq datasets and literature, and (iv) genes linked to Notch/NF-κB signaling. Remarkably, among 131 candidate genes, 103 contained at least one CT-site (Fig. 3d and Supplementary Table S3). Of these, 84 exhibited double *in vivo* dual occupancy by both RBPJ and NF-κB, and 18 were occupied only by NF-κB, and one gene showed RBPJ-only occupancy. The remaining 28 genes lacked CT-sites in which five showed no occupancy by either transcription factor, while the rest showed binding by at least one factor. Importantly, we identified 62 conserved genes in the human genome that harbor CT-sites and also exhibit dual occupancy (Supplementary Table S3). The occurrence of CT-sites in promoter regions of brain-related genes is not based on a simple statistical distribution. Analysis of a set of genes associated with liver, lung, and heart function (Supplementary Table S4) revealed a prominent enrichment of CT-site-containing genes in the group of brain aging-related candidate genes (Supplementary Table S5), arguing for a critical contribution of Notch/NF-κB crosstalk signaling to brain aging.

*Il6* represents a prototypical SASP gene, whereas *Hes1* is a well-known Notch target gene. Both genes are typical representatives carrying CT-sites. *Hes1* possesses two promoter-proximal regions on mouse chromosome 16 with overlapping binding of RBPJ and RelA/NF-κB1, along with chromatin accessibility and active histone marks (Fig. 3e). One key region (chr16:30065000–30065583) contained distinct binding sites for RBPJ, NF-κB, and a composite CT motif. Similarly, the *Il6* promoter region (chr5:30012816–30013411) harbored a CT-site with double occupancy and contained two additional NF-κB sites and one RBPJ-binding site (Fig. 3f). Gene Ontology (GO) analysis of the “brain aging-related” CT gene set with double occupancy by g:Profiler in both mouse and human revealed a strong enrichment in molecular functions (GO:MF) processes related to binding of protein, transcription factors, DNA, receptors, and kinase. Among biological processes (GO:BP), terms associated with metabolism, cellular senescence, stress responses, development, cell death, and immune functions beside others were strongly represented. In the neural context, astrocytic activation and differentiation as well as neuroinflammation and neuronal development were overrepresented as well. For cellular components (GO:CC), candidates of this gene set are present in terms related to membrane rafts and microdomains, various vesicles, and nuclear components. Interestingly, analyses utilizing the KEGG database show a significant enrichment on this gene set in pathways of longevity regulation (KEGG:04 211) and neurodegeneration (KEGG:05 022) both in mice and humans (Supplementary Table S1).

These results establish the composite RBPJ/NF-κB-binding motifs as potential regulatory elements integrating Notch and NF-κB signaling during brain aging.

### Notch signaling selectively inhibits NF-κB/RelA-driven transcription

To investigate the transcriptional consequences of these RBPJ/NF-κB CT-site DNA-binding motifs in the integration of Notch and NF-κB signaling, we employed luciferase reporter assays using established pathway-specific constructs. These include a classical Notch reporter containing 12 tandem RBPJ binding sites [37] and a NF-κB reporter with 3 tandem κB motifs [38], both driving a minimal β-globin promoter upstream of the luciferase gene (Fig. 4a, d).

**Figure 4.**
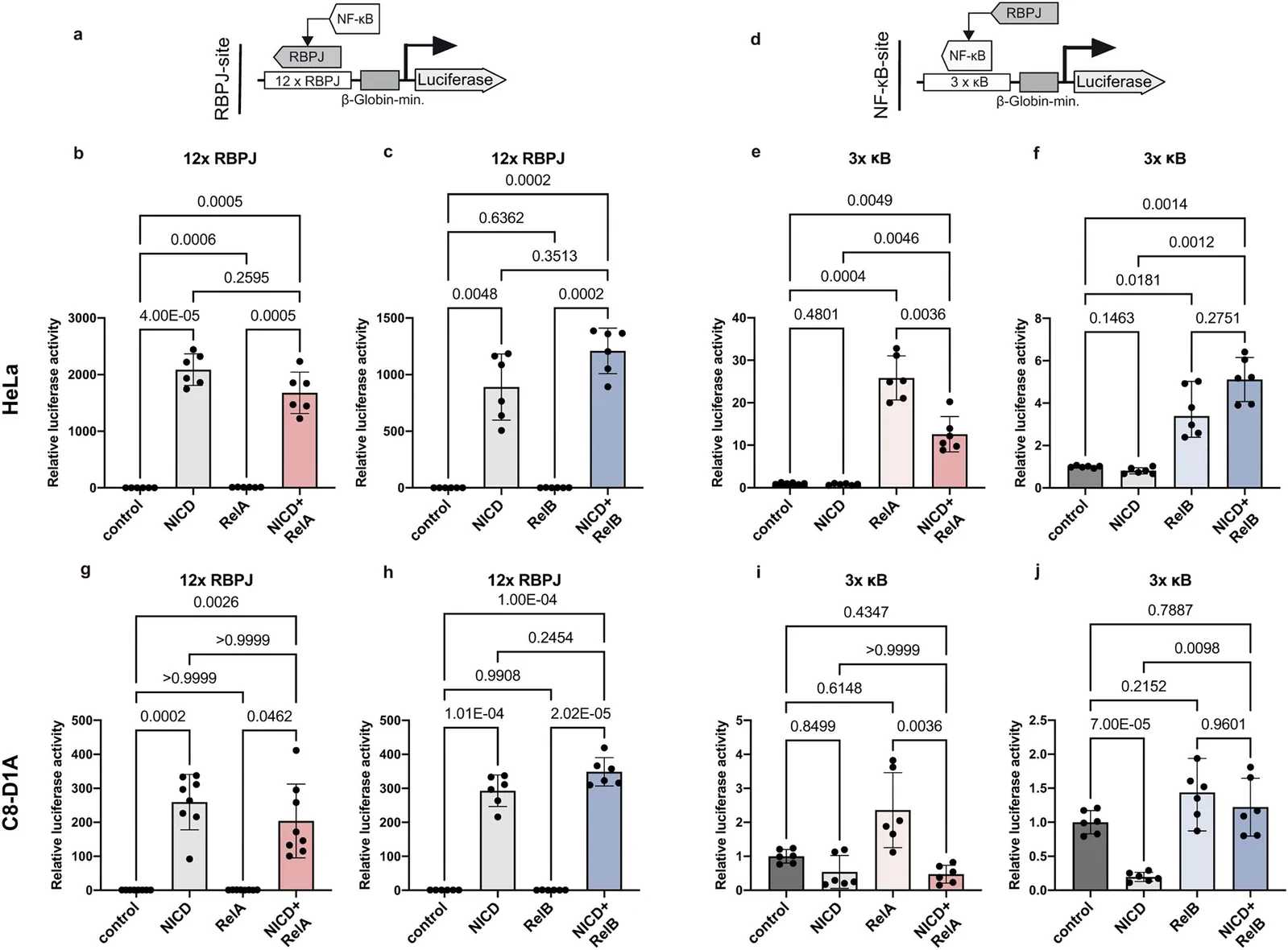
NICD selectively inhibits RelA-driven NF-κB transcriptional activity. (**a, d**) Schematic illustration of the RBPJ-specific reporter and NF-κB-responsive reporter construct. (**b, c, e, f**) Quantification of relative luciferase activity in HeLa cells transiently transfection with 250 ng of 12×RBPJ reporter (b, c) or 3×κB reporter (e, f) together with NICD and individual NF-κB subunits (RelA, RelB), either individually or in combinations (NICD + each NF-κB subunit). (**g, h, i, j**) Quantification of relative luciferase activity in C8-D1A mouse astrocyte cells transiently transfection with 250 ng of 12×RBPJ reporter (g, h) or 3×κB reporter (i, j) together with NICD and individual NF-κB subunits (RelA, RelB), either individually or in combinations (NICD + each NF-κB subunit). Data are shown as mean ± SD from at least three independent experiments. All values were normalized to the basal activity of the reporter construct alone, used as the control. Statistical analysis: non-normally distributed data were analyzed using the Kruskal–Wallis test followed by Dunn’s post-hoc test; for unequal variances, Brown–Forsythe or Welch’s ANOVA with Dunnett’s T3 post-hoc test was used.

We first examined the effects of Notch activation on NF-κB–driven transcription. For this, HeLa cells and C8-D1A, a murine cerebral astrocyte cell line were co-transfected with the κB reporter and expression plasmids encoding NICD (activated Notch intracellular domain) and the NF-κB subunits RelA and RelB, representing the canonical and alternative NF-κB pathway. In HeLa cells, RelA induced the strongest NF-κB reporter activity, approximately 5-fold higher than RelB (Fig. 4e, f). NICD alone did not activate the NF-κB reporter; however, co-expression of NICD with RelA significantly suppressed RelA-induced NF-κB reporter activity. In contrast, co-expression of NICD with RelB does not affect RelB-driven NF-κB activation. Expression of the overexpressed constructs remained comparable under the corresponding co-transfection conditions, excluding reduced protein expression as an explanation for the observed changes in reporter activity (Supplementary Fig. S5a). Importantly, the selective inhibition of RelA-driven NF-κB transcription by NICD was also observed in C8-D1A astrocytes (Fig. 4i, j).

To determine whether NF-κB signaling reciprocally regulates Notch-dependent transcription, we assessed NICD-induced activation of the 12×RBPJ reporter in the presence or absence of NF-κB subunits. In both HeLa cells and C8-D1A astrocytes, NICD alone induced strong reporter activity, whereas expression of RelA and RelB failed to do so (Fig. 4b, c, g, h). However, co-expression of any NF-κB subunit together with NICD was not able to interfere with NICD/RBPJ-induced transcription, implying only a one-sided interaction of Notch signaling on NF-κB-dependent gene expression.

### Notch signaling differentially modulates NF-κB–dependent transcription of SASP genes harboring RBPJ/NF-κB CT-sites

To assess the function of the newly identified RBPJ/NF-κB CT-sites in transcriptional regulation, we cloned CT-sites from *Hes1, Il6*, and additional SASP-related genes (*Ifnb1, Igk, Lcn2, Cdr1*, and *Tnip1*) into luciferase reporter constructs, each containing three tandem CT repeats upstream of a minimal β-globin promoter and the luciferase reporter gene (Fig. 5a, c, f). The *Hes1, Il6*, and *Lcn2* CT-site reporter genes were activated by both NICD and RelA in HeLa cells, although with distinct activation patterns. *Hes1* was more responsive to NICD, while *Il6* and *Lcn2* were more prominently activated by RelA. Notably, co-expression of NICD with RelA did not lead to cooperation but significantly suppressed reporter activity from those constructs compared to individual NICD or RelA-mediated activation (Fig. 5b, d, e). Similar inhibition of reporter activity was observed in C8-D1A astrocytes following co-transfection with NICD and RelA compared with their respective individual responsive constructs (Supplementary Fig. S5c, e).

**Figure 5.**
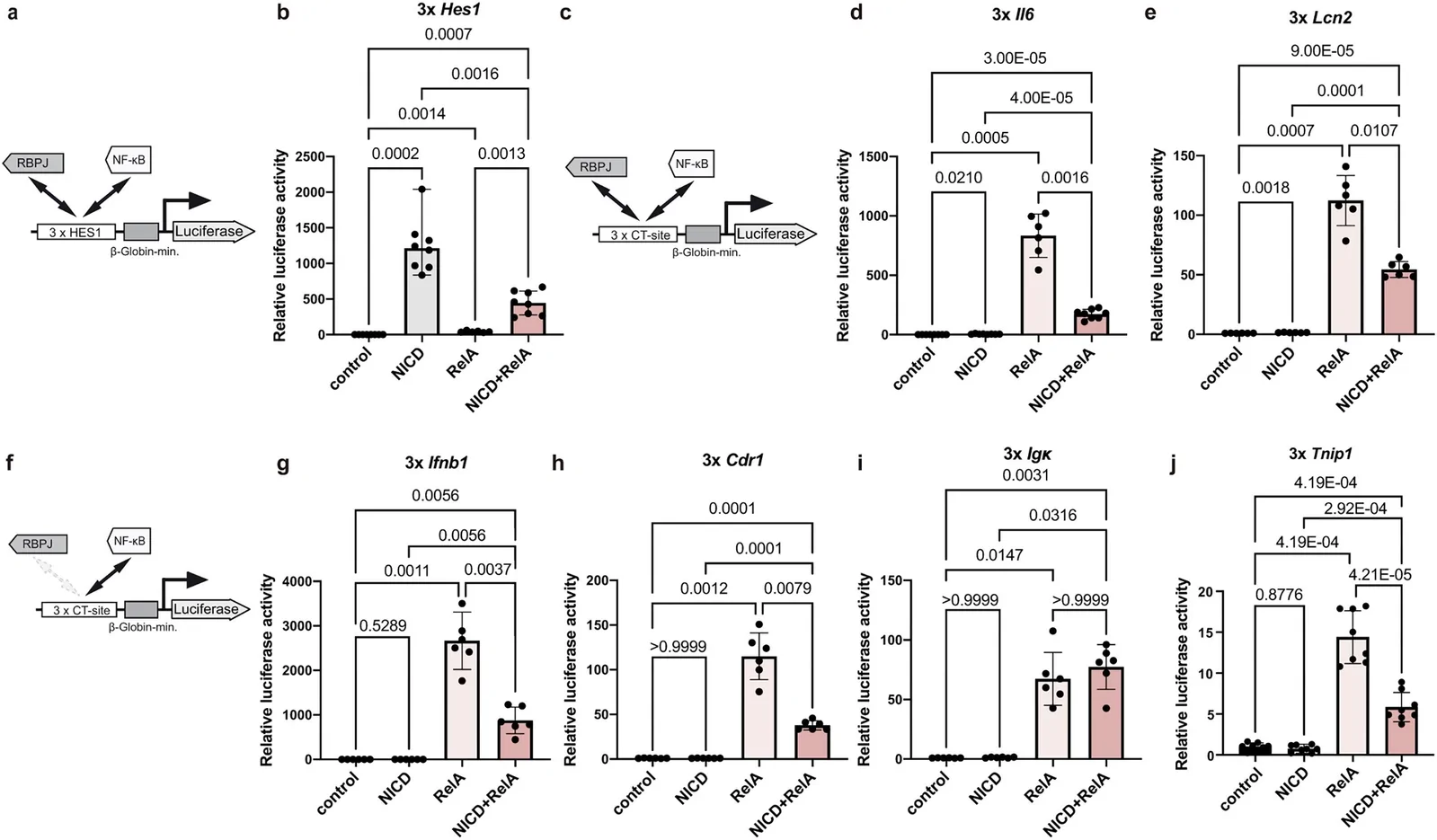
Functional assessment of RBPJ/NF-κB CT-sites from SASP genes. (**a, c, f**) Schematic representation of the 3×CT-site reporter constructs for *Hes1, Il6, Lcn2, Ifnb1, Cdr1, Igk*, and *Tnip1*. (**b, d, e, g–j**) Quantification of relative luciferase activity from *Hes1* and other SASP-related CT-site (*Il6, Lcn2, Ifnb1, Cdr1, Igk*, and *Tnip1*) reporters in response to NICD, RelA, or their co-expression. HeLa cells were transiently co-transfected with 250 ng of the 3×CT-site reporter construct and 20 ng of expression plasmids encoding NICD, RelA, or both. Data represent mean ± SD from at least three independent experiments. All values were normalized to the basal activity of the reporter construct alone, used as the control. Statistical analysis: non-normally distributed data were analyzed using the Kruskal–Wallis test followed by Dunn’s post-hoc test; for unequal variances, Brown–Forsythe or Welch’s ANOVA with Dunnett’s T3 post-hoc test was applied.

When we analyzed the other CT-site reporter gene constructs, we observed selective responsiveness to RelA in reporters derived from *Ifnb1, Cdr1, Igk*, and *Tnip1* (Fig. 5g, h, i, j). The *Ifnb1* reporter was strongly induced by RelA, and NICD co-expression markedly suppressed this activation (Fig. 5g). *Cdr1* showed moderate RelA responsiveness and was similarly inhibited by NICD (Fig. 5h). In contrast, *Igk* and *Tnip1* displayed only modest RelA-mediated activation, with NICD having no significant effect on *Igk* but significantly inhibiting the reporter activity of *Tnip1* (Fig. 5i, j).

These findings suggest that activated Notch signaling can differentially modulate NF-κB-mediated transcriptional activation at specific gene loci containing CT and/or NF-κB sites, likely through competitive binding or protein–protein interference at shared promoter elements.

### NICD-mediated suppression of RelA/NF-κB–dependent transcription of CT genes occurs at different levels

To elucidate the molecular basis of the Notch and NF-κB crosstalk, we examined the physical interaction between NICD and core NF-κB subunits at the protein level. Co-immunoprecipitation (Co-IP) assays were conducted in HEK293 cells co-expressing NICD with either RelA or RelB. NICD exhibited a robust interaction with RelA, as confirmed by both forward and reverse Co-IP (Fig. 6a, b). In contrast, no detectable interaction was observed between NICD and RelB (Fig. 6a).

**Figure 6.**
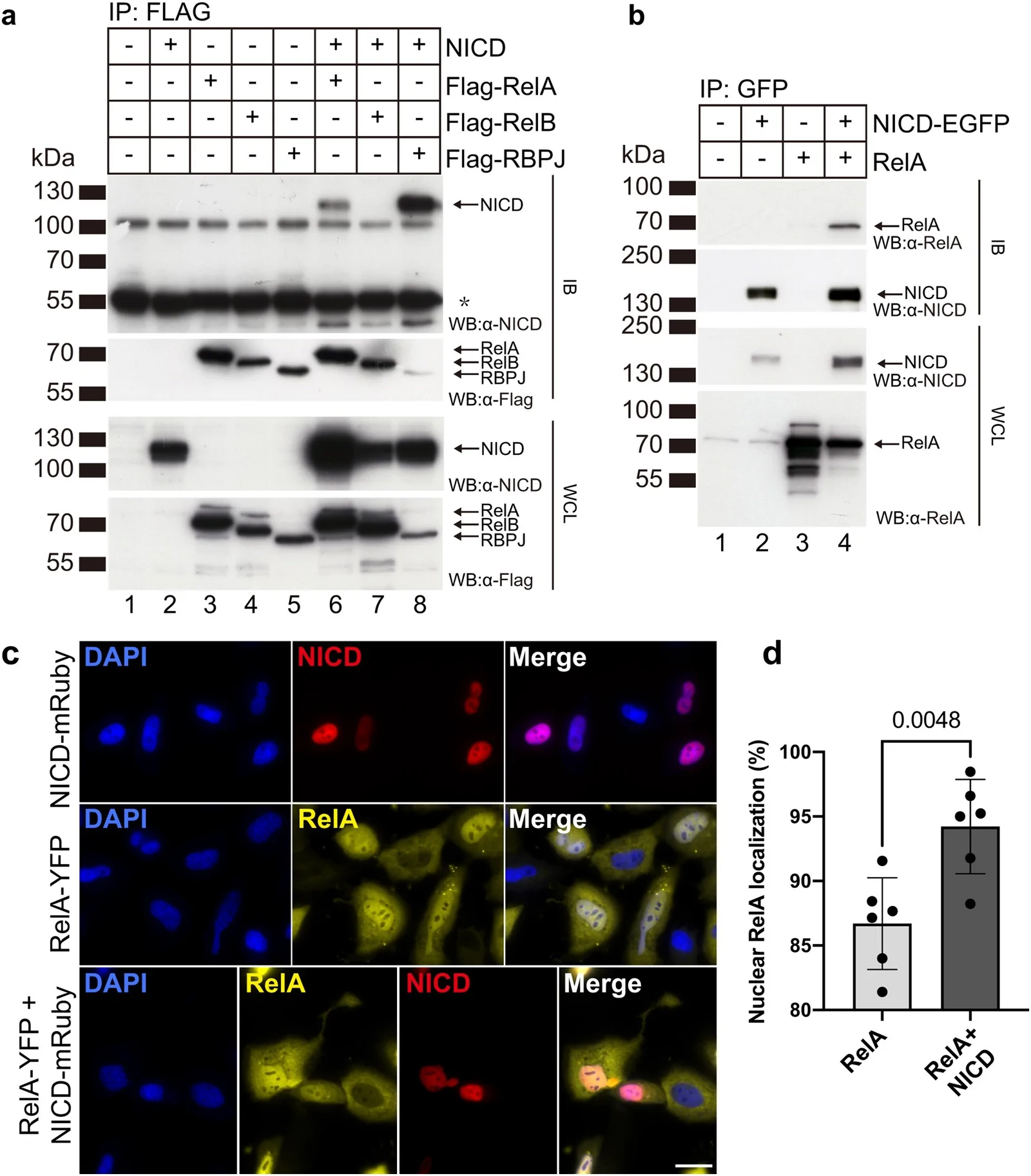
Physical interaction and nuclear co-localization of NICD with NF-κB subunits. (**a**) Co-IP of NICD with RelA and RelB using anti-Flag antibody-conjugated beads. Western blots were performed with either anti-NICD (Abcam Limited, Cat. no. ab128076) or anti-Flag antibody. (**b**) Co-IP of NICD with RelA using anti-GFP antibody and Protein G beads. Western blots were probed with either anti-NICD (Abcam Limited, Cat. no. ab128076) or anti-RelA antibody. HEK293 cells were co-transfected with 10 μg of the corresponding expression plasmids 24 h prior to harvest. Anti-Flag antibody-conjugated beads were used to immunoprecipitate Flag-tagged proteins and their binding partners. Flag-RBPJ co-transfected with NICD served as a positive control. For GFP-tagged proteins, an anti-GFP antibody and Protein G beads were used for pulldown. 20 μl of IP samples were loaded for western blot analysis, while 10 μg whole-cell lysate (WCL) from each sample was loaded as input. (−) indicates absence of the expression construct; (+) indicates protein expression. The top panels display western blot results of Co-IP experiments to assess protein–protein interactions. The second panels show the control IP samples analyzed by western blot to confirm the efficiency of immunoprecipitation. Both lower panels show the expression control of the respective interaction partners in WCLs. Specific immunoreactive bands are marked with arrows. (*) denotes heavy chains of the anti-Flag antibody. (**c**) Subcellular co-localization of NICD and RelA. NICD exhibited predominant nuclear localization, whereas RelA was distributed in both the cytoplasm and nucleus. (**d**) Quantification of co-transfection resulted in prominent nuclear co-localization of NICD and RelA. HeLa cells were transfected with 200 ng of NICD-mRuby and RelA-YFP, either individually or in combination. Nuclear DNA was counterstained with DAPI (left column). Fluorescent signals from mRuby or YFP are shown in the middle column, and merged images are presented in the right column. Images were acquired using a fluorescence microscope equipped with a 60× objective. Scale bars represent 10 μm. Statistical analysis: unpaired two-tailed Student’s *t*-test.

To determine the subcellular distribution and potential co-localization of NICD and RelA, we performed immunofluorescence staining in HEK293 cells co-transfected with NICD-mRuby and RelA-YFP (Fig. 6c). When expressed individually, NICD exhibited a strong nuclear localization, while RelA was distributed throughout the cell but showed pronounced nuclear enrichment. In co-transfected cells, NICD and RelA displayed clear nuclear co-localization, with additional overlapping signals observed at the nuclear periphery. Quantitative analysis further revealed that co-expression of NICD significantly increased the nuclear enrichment of RelA compared with RelA expression alone (Fig. 6d). Together, these findings support a potential nuclear interplay between activated Notch and RelA/NF-κB at the level of gene transcription.

To further dissect the molecular mechanism by which NICD modulates RelA/NF-κB transcriptional activity at CT-site-containing loci, we investigated whether DNA-binding capability of RelA is required for NICD-mediated inhibition. We employed both wild-type RelA (RelAwt) and a DNA-binding–defective point mutant (RelA–E39I) in luciferase reporter assays using 3×*Il6*, 3×*Hes1*, and 3×κB reporters. To distinguish the contribution of exogenous RelA activity from potential interference by endogenous NF-κB signaling, reporter assays were performed in both HeLa-RelA^wt^ and HeLa-RelA^KO^ cells (Fig. 7a–c, d–f). In a control immunofluorescence experiment using RelA-knockout HeLa cells, the RelA–E39I mutant, similar to RelAwt, still shows nuclear accumulation 30 min after TNF-α treatment (Fig. 7g) ruling out that decreased transactivation capacity of RelA–E39I is due to failure of TNF-α responsiveness. In the reporter gene assays, expression of either RelAwt or NICD was able to strongly activate the 3×*Hes1* reporter gene, whereas RelA–E39I failed to do so. Interestingly, co-expression of NICD with RelA–E39I together with the 3×*Hes1* reporter resulted in an even stronger inhibition of NICD-driven transcription than co-expression with RelAwt, even in the absence of endogenous RelA (Fig. 7b, e). In case of the *Il6* CT-site reporter and the classical 3×κB reporter, which are rather NF-κB sensitive, RelAwt induced prominent activation, while RelA–E39I only activated at a low level (Fig. 7a, c, d, f). NICD co-expression significantly repressed RelAwt-induced transcription of the 3×*Il6* reporter, whereas the low level RelA–E39I-driven activity was only mildly affected. Again, the 3×κB reporter was not transactivated by NICD (Fig. 7c, f) These results implicate that the inhibitory effect of NICD on RelA-mediated CT-site reporter gene activation persists in the absence of endogenous RelA, suggesting that this inhibition involves at least two mechanisms: a DNA-binding-dependent mechanism involving CT-sites and protein–protein interaction. Vice versa, the repression of NICD-induced *Hes1* CT-site reporter activity, which is primarily Notch sensitive, likely relies only on protein–protein interaction with RelAwt or RelA–E39I independent of any DNA-binding abilities. In line with this, Co-IP experiments showed that RelA–E39I retained the ability to physically interact with NICD, although the interaction was slightly weaker than with RelAwt (Fig. 7h). Collectively, these findings provide strong evidence that Notch-mediated inhibition of NF-κB–dependent transcription at CT-site gene loci bases on a dual mechanism including interference at CT-sites and direct NICD–RelA interaction.

**Figure 7.**
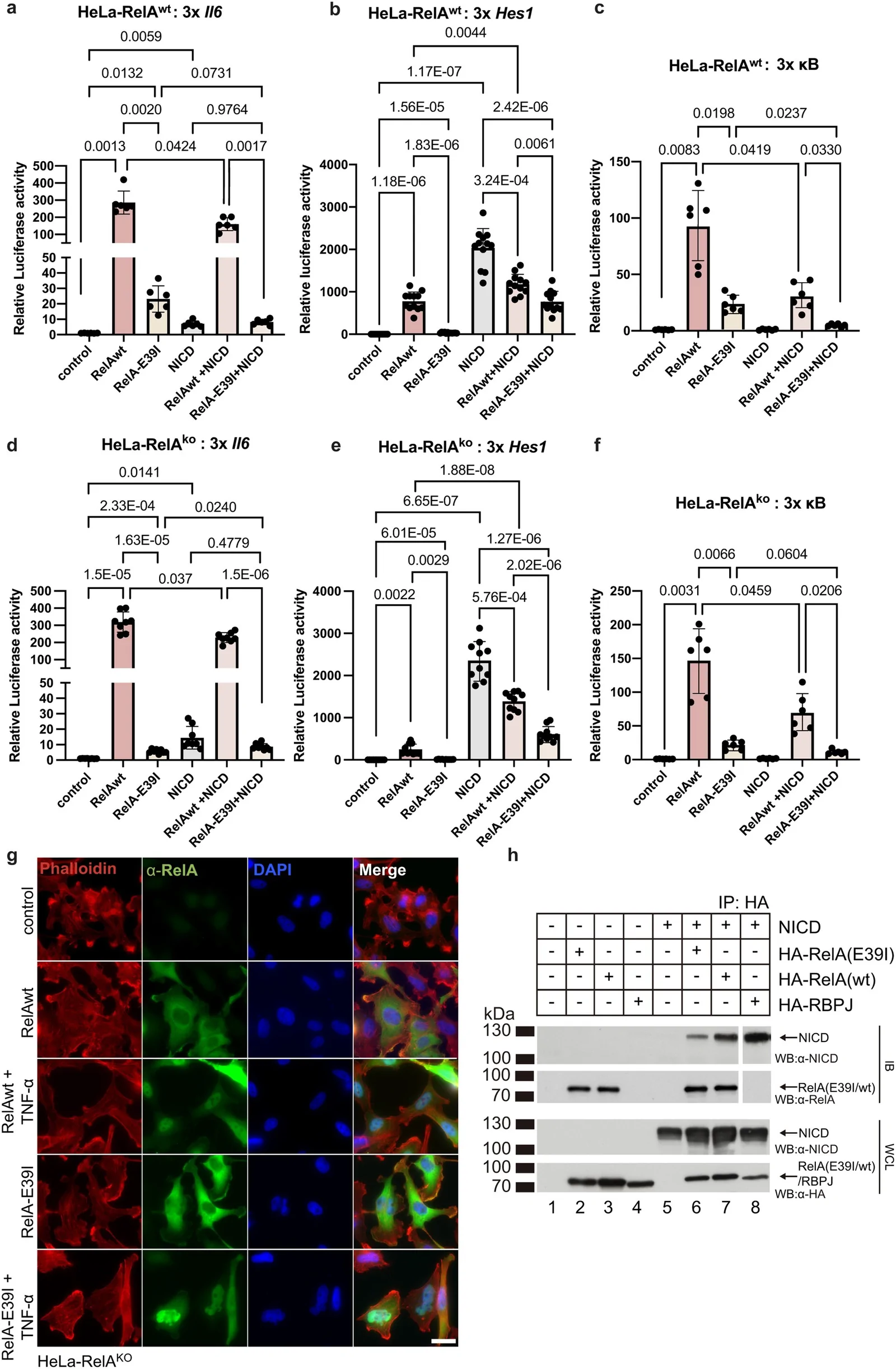
Assessing Notch modulation of NF-κB–dependent transcription of SASP genes using DNA-binding–defective RelA mutant. (**a, b, c**) Quantification of relative luciferase activity from 3×*Il6* (a), 3×*Hes1* (b), CT-site reporter, and 3×κB (c) reporter in response to wild-type RelA, DNA-binding–defective RelA mutant (RelA–E39I) or their co-expression with NICD in HeLa-RelA^wt^ (HeLa with RelA^wt^ status) cells. (**d, e, f**) Quantification of relative luciferase activity from 3×*Il6* (d), 3×*Hes1* (e), CT-site reporter, and 3×κB (f) reporter in response to wild-type RelA, DNA-binding–defective RelA mutant (RelA–E39I), or their co-expression with NICD in HeLa-RelA^ko^ (HeLa with RelA^ko^ status) cells. Cells were transiently co-transfected with 250 ng of the indicated luciferase reporter plasmids and 20 ng of expression plasmids encoding NICD, RelAwt, or RelA–E39I, alone or in combination. Data are presented as mean ± SD from at least three independent experiments. All values were normalized to the basal activity of the reporter construct alone, used as control. Statistical analysis: Brown–Forsythe or Welch’s ANOVA with Dunnett’s T3 post-hoc test was applied. (**g**) Immunofluorescence analysis of HeLa-RelA^KO^ cells reconstituted with RelAwt or the DNA-binding–defective mutant RelA–E39I, either untreated or stimulated with TNF-α (20 ng/ml) for 30 min. Nuclear DNA was counterstained with DAPI. Cells were stained for RelA and actin using anti-RelA antibody and Alexa Fluor^®^ 568 conjugated phalloidin. Images were acquired using a fluorescence microscope equipped with a 60× objective. Scale bars represent 10 μm. (**h**) NICD showed interaction with both RelA–E39I (lane 6) and RelAwt (lane 7). HEK293 cells were co-transfected with 10 μg of the respective expression plasmids and harvested after 24 h. Proteins were immunoprecipitated using an anti-HA antibody and Protein G beads. HA-RBPJ co-transfected with NICD served as a positive control. Western blotting was performed with anti-NICD (Abcam, Cat. no. ab128076) and anti-HA antibodies. Input lanes represent 10 μg of total lysate (WCL), and 20 μl of immunoprecipitate was loaded for each sample. (−) indicates absence of the expression construct; (+) indicates protein expression. The top panels show western blot results of Co-IP experiments assessing protein–protein interactions. The second panels show the control IP samples analyzed by western blot to confirm the efficiency of immunoprecipitation. Both lower panels show the expression control of the respective interaction partners in WCLs. Specific immunoreactive bands are indicated by arrows.

### NICD preferentially interacts with activated IKK2 resulting in mutual inhibition of Notch and NF-κB signaling

Our studies revealed that RelB failed to interact with NICD whereas RelA showed a robust interaction arguing for a selective connection of NICD with the canonical, inflammatory IKK/Nemo/NF-κB signaling pathway. To further address this issue, we investigated how NICD engages upstream signaling components of the canonical pathway. Here, we focused on IKK2, the primary catalytic kinase in this pathway and performed Co-IP assays using well-characterized IKK2 mutants representing distinct phosphorylation or catalytic states. The constitutively active (IKK2-CA) mutant was generated by substituting serine residues at positions 177 and 181 with glutamic acid (S177E/S181E), mimicking phosphorylation and rendering the kinase active independent of upstream signaling. The phosphorylation-deficient mutant (AA, S177A/S181A) prevents activation through phosphorylation, while the kinase-dead mutant (DN, D145N) disrupts ATP binding and catalytic activity. The positions of these mutations were highlighted on the human IKK2 crystal structure (PDB ID: 4KIK) visualized in PyMOL (Fig. 8a). NICD exhibited minimal or no binding to the DN and AA mutants, with no appreciable enhancement upon TNF-α treatment (Fig. 8b, c). In contrast, IKK2-CA exhibited robust interaction with NICD, further enhanced by TNF-α stimulation (Fig. 8d). IKK2-WT displayed a moderate interaction with NICD under basal conditions, which was markedly enhanced after TNF-α treatment (Fig. 8e). These results indicate that NICD preferentially associates with the active form of IKK2 *in vitro*.

**Figure 8.**
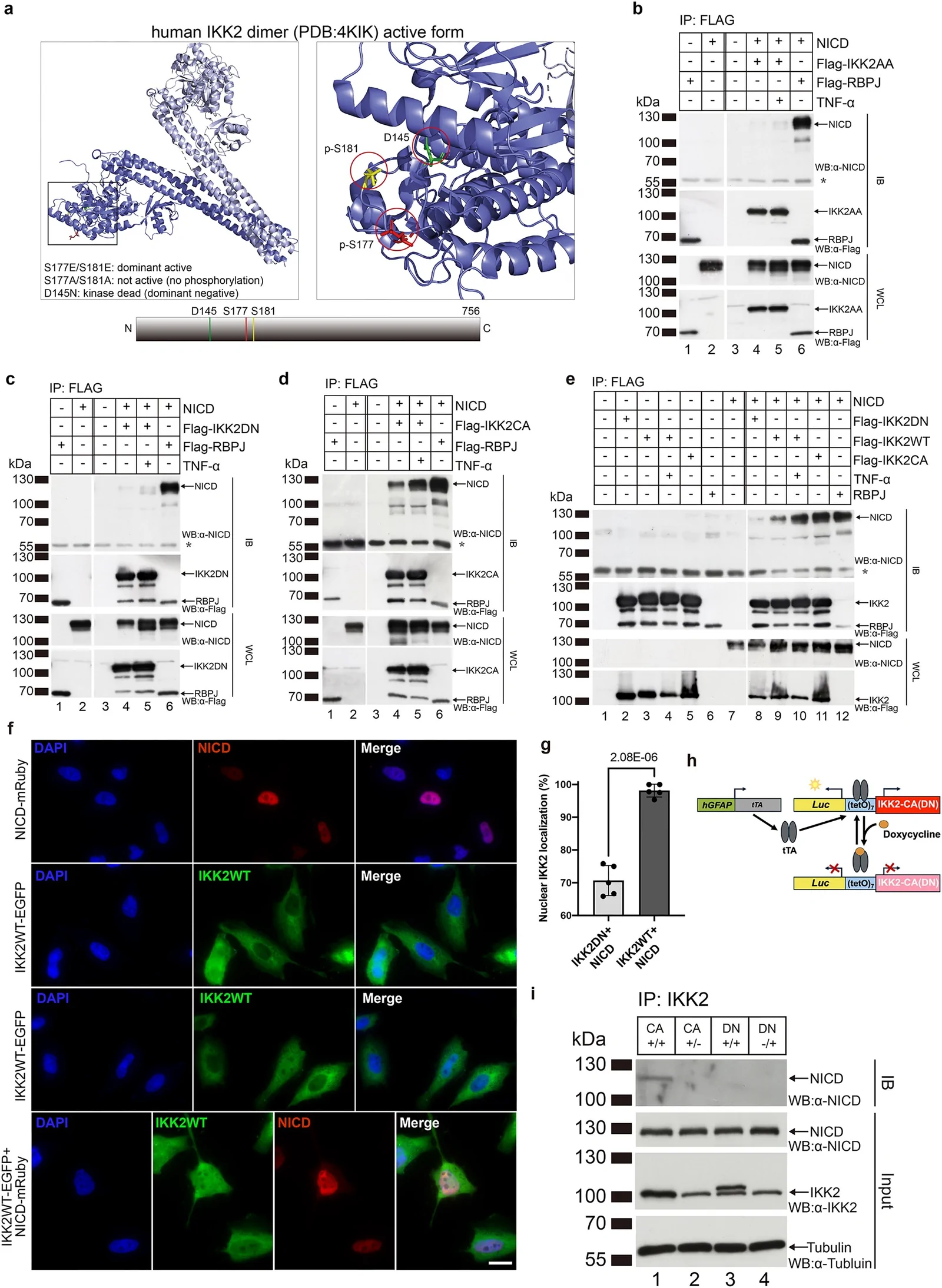
NICD preferentially interacts with active IKK2. **(a)** Crystal structure of the human IKK2 dimer (PDB: 4KIK) visualized using PyMOL. A zoomed-in view (right panel) highlights key residues D145 (green), S177 (red), and S181 (yellow), which were mutated to generate the DN (D145N), CA (S177E/S181E), and AA (S177A/S181A) variants, respectively. A schematic diagram illustrates the domain architecture and mutation sites within the full-length IKK2 (bottom panel). (**b**–**d**) Co-IP of NICD with IKK2-AA (b), IKK2-DN (c), IKK2-CA (d), and with or without TNF-α. (**e**) Comparative analysis of NICD interaction with IKK2 variants (DN, WT, WT + TNF-α, CA). HEK293 cells were co-transfected with 10 μg NICD and 10 μg various Flag-tagged IKK2 constructs (AA, DN, WT, and CA). Where indicated, cells were stimulated with TNF-α (50 ng/ml) for 30 min, starting 22 h after transfection, prior to lysis to activate IKK2. Protein complexes were immunoprecipitated using anti-Flag antibody-conjugated beads. Flag-RBPJ co-transfected with NICD served as a positive control. 20 μl of immunoprecipitated samples were loaded for western blot analysis, while 10 μg WCL from each sample was loaded as input to verify protein expression. Blots were probed with anti-NICD and anti-Flag antibodies as indicated. (−) indicates absence of the construct; (+) indicates presence. The top panels show Co-IP results, with second panels displaying control IP blots and the lower panels confirm expression of input proteins in WCLs. Specific immunoreactive bands are indicated by arrows. (*) denotes heavy chains of the anti-Flag antibody. (**f, g**) Subcellular co-localization (f), and quantification (g) of NICD with IKK2. HeLa cells were transfected with 200 ng of NICD-mRuby and IKK2-WT-EGFP, either individually or together. Nuclei were counterstained with DAPI (left column). Fluorescent signals from mRuby or EGFP are shown in the middle column, and merged images are presented in the right column. NICD was localized in the nucleus (first panel), while IKK2WT was distributed in both nucleus and cytoplasm (second and third panel). Co-expression revealed prominent nuclear co-localization of NICD with IKK2-WT (fourth panel). Images were acquired using a fluorescence microscope (60× objective). Scale bars:10 μm. Statistical analysis: unpaired two-tailed Student’s *t*-test. (**h**) Schematic illusion of transgenic mouse models expressing IKK2-CA or IKK2-DN under the control of a tetracycline-inducible system. (**i**) Co-IP of NICD interactions with IKK2 mutants *in vivo*. Cortical lysates were collected from heterozygous (+/−) and homozygous (+/+) mice (17–19 weeks), expressing either IKK2-CA or IKK2-DN variants. Co-IP was performed using anti-IKK2 antibody to pull down endogenous IKK2 and interaction partners. IP samples (20 μl) and WCL (30 μg) were analyzed by western blot using anti-NICD and anti-IKK2 antibodies. Top panel: NICD (~130 kDa) was detected only in IKK2-CA^GFAP^ (+/+) samples. Bottom panel: Input blot confirming comparable NICD and IKK2 levels in cortex lysates. Tubulin was used as a loading control to ensure equal protein input. Arrows indicate specific protein bands.

To validate these findings *in vivo*, we performed co-IP assays using cortical lysates from transgenic mice with astrocyte-specific expression of dominant active (IKK2-CA^GFAP^) or dominant-negative (IKK2-DN^GFAP^) IKK2 (Fig. 8h, i). Endogenous NICD was specifically detected in immunoprecipitates from IKK2-CA^GFAP^ mice, but not from IKK2-DN^GFAP^ or control mice (Fig. 8i). The IKK complex is usually located in the cytoplasm where it gets activated by upstream kinases. Fluorescence staining confirmed that IKK2 displayed mainly a cytoplasmic distribution. Notably, co-expression of NICD increased the nuclear enrichment of IKK2, as confirmed by quantitative analysis (Fig. 8f, g), thus providing additional starting points for functional interference.

Functionally, co-expression of NICD with either IKK2-WT or IKK2-CA significantly suppressed IKK2/NF-κB-driven transcriptional activity in HeLa cells. Importantly, this inhibitory effect was recapitulated in C8-D1A astrocytes, indicating that NICD acts as a negative modulator of NF-κB signaling (Fig. 9a, b, e, f). However, in contrast to the effects observed with RelA co-expression (Fig. 4b, g), co-expression of IKK2-CA (Fig. 9c, g), and IKK2-WT (Fig. 9d, h) substantially suppressed Notch-dependent transcription, with more pronounced inhibitory effect observed with IKK2-CA. However, co-expression of IKK2-AA (Supplementary Fig. S6a) and IKK2-DN (Supplementary Fig. S6b) did not suppress Notch-dependent transcription. Together, these findings imply a reciprocal inhibitory interaction between Notch and activated IKK2/NF-κB signaling.

**Figure 9.**
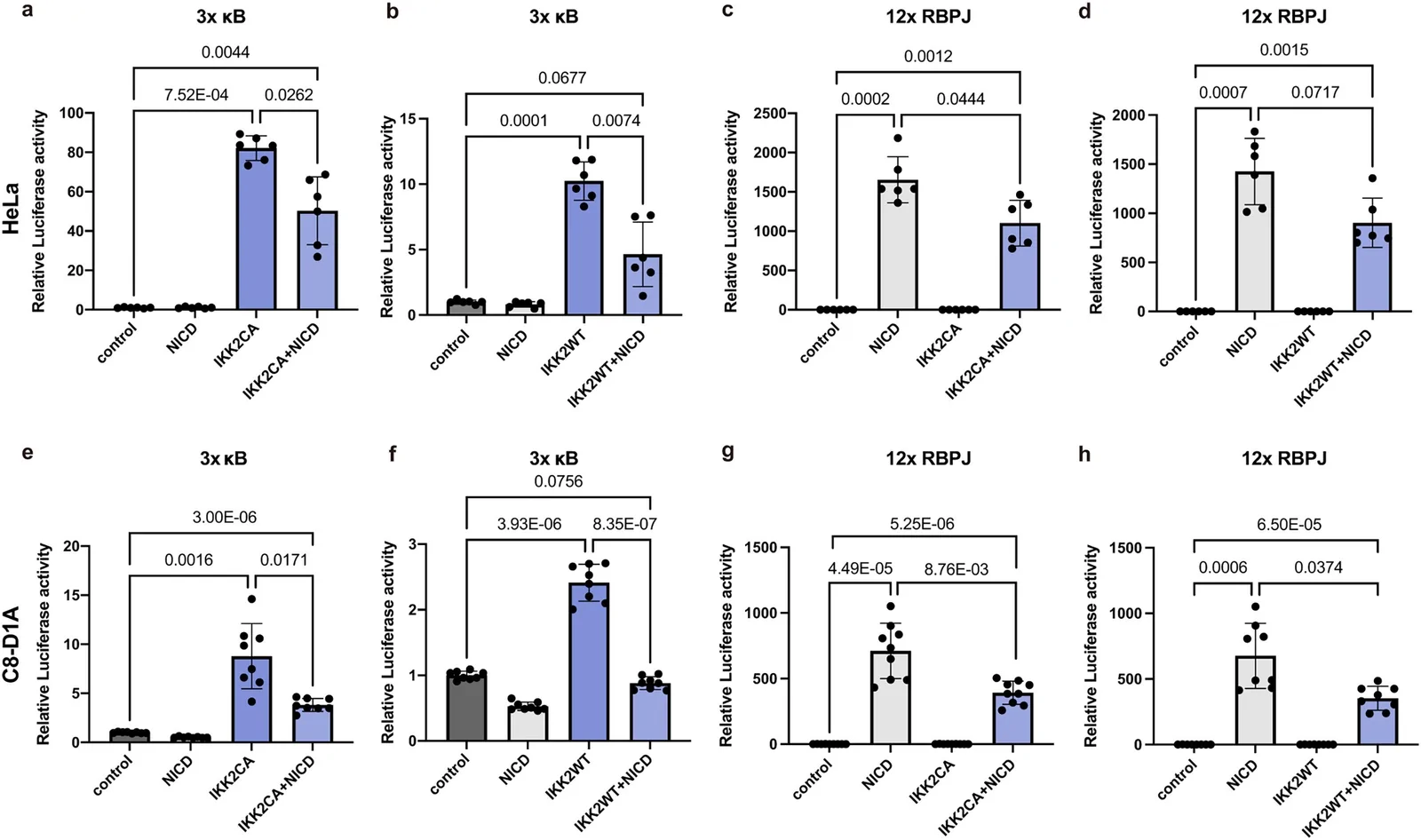
Reciprocal modulation of Notch and NF-κB transcriptional activity by NICD and IKK2. (**a**–**h**) Reciprocal modulation of Notch and NF-κB transcriptional activity by NICD and IKK2 in HeLa (a–d) and C8-D1A mouse astrocyte (e–h) cells. Cells were transiently co-transfected with 250 ng of either the 3×κB or 12×RBPJ luciferase reporter and 20 ng NICD, IKK2-CA, IKK2-WT, IKK2-AA, or IKK2-DN expression plasmids, alone or in combination. Data represent mean ± standard deviation from at least three independent experiments, each performed with two technical replicates. All values were normalized to the reporter-only control. Statistical analysis: Brown–Forsythe or Welch’s ANOVA, followed by Dunnett’s T3 post-hoc test was used. Panels a, b, e, and f: 3×κB reporter; Panels c,d, g, and h: 12×RBPJ reporter. Panels a, c, e, and g: IKK2-CA; panels b, d, f, and h: IKK2-WT.

### NICD interacts with IKK1 regardless of activation status and cooperatively modulates NF-κB transcriptional activity with IKK2

Since we found this specific interaction of NICD with activated IKK2, we also examined IKK1, the second kinase within the IKK complex. Similar to our experiments with IKK2, we co-expressed IKK1 variants (WT, CA, AA, and DN) with NICD in HEK293 cells. The CA mutant (S176E/S180E) mimics phosphorylation at activation loop serine residues, while the AA mutant (S176A/S180A) prevents activation by blocking phosphorylation. The DN variant (K44M) abolishes ATP binding, rendering the protein catalytically inactive (Supplementary Fig. S7a). Co-IP analysis revealed that NICD robustly co-precipitated with all IKK1 variants, suggesting that the NICD–IKK1 interaction is independent of the IKK1 activation status (Supplementary Fig. S7b).

To further explore the composition of the precipitated complexes, we probed for other endogenously expressed components of the IKK complex, IKK2 and NEMO. Interestingly, stronger endogenous IKK2 signals were detected in all IKK1 precipitates together with NICD co-transfected groups compared to IKK1-only control, regardless of the IKK1 variant used (Supplementary Fig. S7b). In contrast, the level of endogenous NEMO remained relatively unchanged upon NICD co-expression. We also examined the presence of RelA and cRel in the IP samples. Here, co-expression of NICD enhanced endogenous RelA co-precipitation across all IKK1 variants, whereas cRel levels were reduced.

To determine the functional consequence of the NICD–IKK1 interaction, luciferase reporter assays were conducted again in HeLa cells. NICD significantly inhibited IKK1-WT and IKK1-CA induced NF-κB activation (Supplementary Fig. 7c, d), although a significant reciprocal effect on Notch transcription was not observed (Supplementary Fig. 7e, f). Consistent with our previous data, subcellular localization of IKK1 was again enriched in the nucleus upon NICD co-expression (Supplementary Fig. S7g).

Together, these results support the view that the NICD–IKK1 interaction is rather indirect and conveyed through a component of the canonical pathway, most probably by activated IKK2. However, both IKK1 and IKK2 show increased nuclear translocation in the presence of NICD.

### Pharmacological Notch activation suppresses NF-κB-driven inflammatory and SASP transcriptional programs in primary brain-derived glial cells

Our findings imply that physical interaction of NICD with activated IKK2 and RelA, central components of the canonical inflammatory NF-κB pathway, may offer an entry point for differential transcriptional regulation of NF-κB-driven SASP gene expression. This may also particularly affect those genes which contain CT-sites in their promotor region. To further investigate the reciprocal relationship between these pathways in a brain-relevant cellular context, we examined transcriptome-wide responses to NF-κB and Notch pathway manipulation in primary brain-derived glial cells.

Thus, brains of adult wild type mice were dissociated using the GentleMACS system. Cells were subsequently labelled with ACSA-2^+^ microbeads, and mixed glia cells (myeloid/microglia/oligodendrocytes) were eluted, cultured, and treated with an NF-κB activation cocktail (TNF-α, poly I:C, LPS), with or without the Notch agonist RIN1 [39]. Subsequent RNA sequencing revealed distinct clustering of the treatment groups in the PCA, indicating differential transcriptional responses (Fig. 10a). Activation with the NF-κB cocktail induced significant upregulation of KEGG terms associated with inflammation and NF-κB (Supplementary Fig. S8a). GSEA further demonstrated significant enrichment of both CT-containing genes and SASP genes lacking CT motifs (MM16098) (Supplementary Fig. S8b, c), whereas the Notch signaling pathway (GO:0 007 219) was markedly downregulated (Supplementary Fig. S8d). These findings indicate that NF-κB activation is associated with induction of inflammatory and SASP transcriptional programs concomitant with suppression of Notch signaling in primary astrocytes.

**Figure 10.**
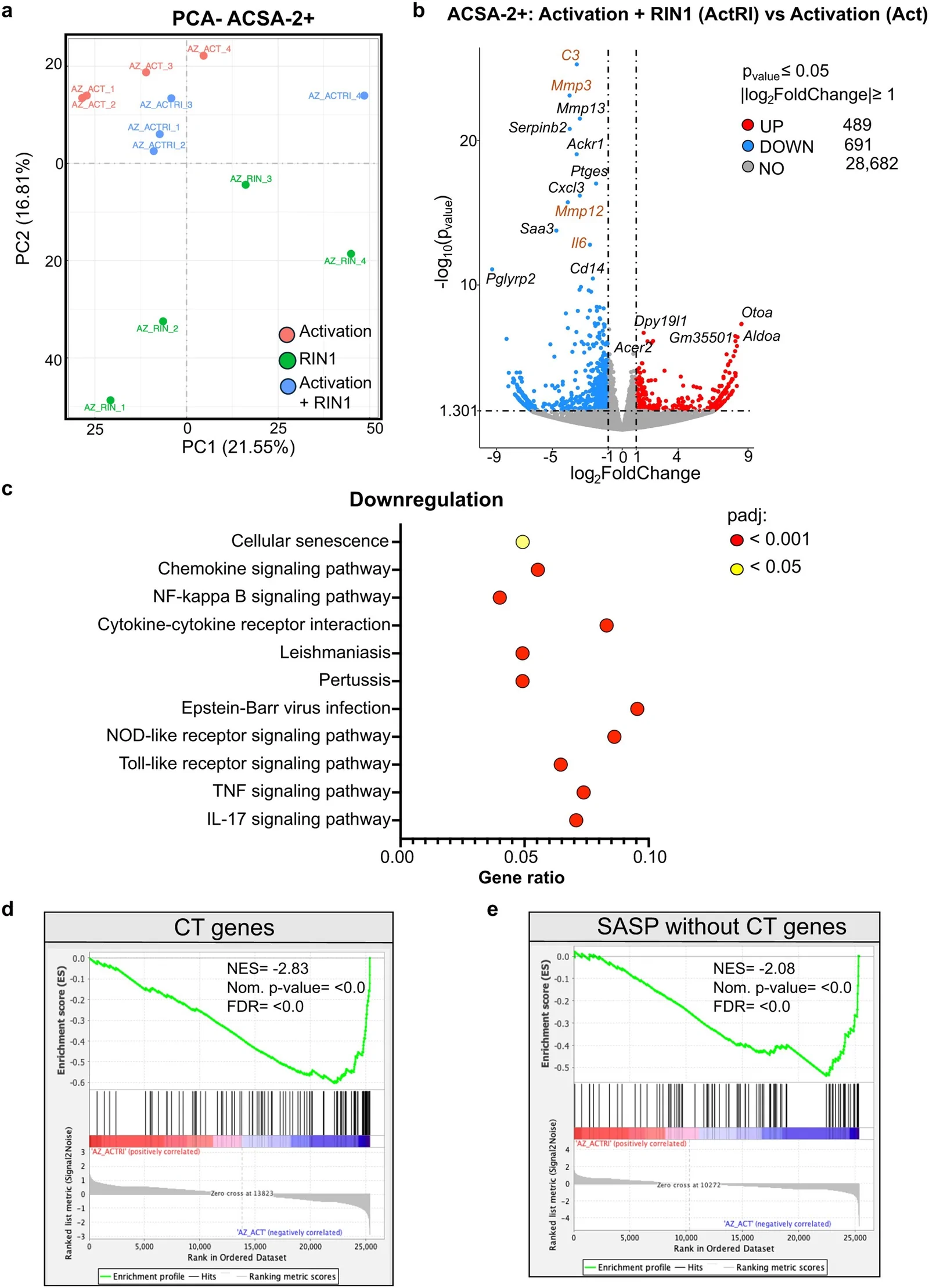
Pharmaceutical Notch activation attenuates NF-κB-driven inflammatory response and SASP development in astrocytes. Primary ACSA-2^+^ astrocytes and flowthrough cells (FT) from 14 to 16 weeks old wild-type mice were taken into culture and treated (16 h) with the Notch agonist RIN1, activation cocktail, or a combination of both, with DMSO as a negative control. After harvest and RNA purification, RNA sequencing was conducted. (**a**) PCA of the treatment groups revealed specific transcriptomic clustering. (**b**) Volcano plot detected downregulation of CT-containing genes (highlighted in red), and (**c**) KEGG enrichment analysis revealed significant downregulation of several terms associated with inflammation and immune activation after co-treatment with RIN1. GSEA analyses confirmed a significantly decreased expression of (**d**) CT genes as well as (**e**) SASP genes without genes containing CT motifs as indicated. Analysis of RNA sequencing by DESeq2 (two-sided Wald’s test), |log2FoldChange| > 1, *P*-value < 0.05. FDR correction of *P*-values (padj) by multiple comparisons test. Over- or underrepresentation of gene sets (GSEA analysis) identified by a gene set-based permutation test and subsequent multiple hypothesis testing.

Comparative analysis with the combined treatment group identified extensive transcriptional changes, including altered expression of multiple CT-containing genes (Fig. 10b). Consistent with the attenuation of the inflammatory response, co-treatment with RIN1 dampened activation of various KEGG terms associated with NF-κB signaling as well as cellular senescence (Fig. 10c). GSEA further demonstrated significant negative enrichment of CT-containing genes (NES = −2.83) and SASP genes lacking CT motifs (NES = −2.08; based on MM16098) upon RIN1 co-treatment (Fig. 10d, e). qRT-PCR analyses of the primary cells, recapitulated the upregulation of selective SASP genes (*C3, Il1b, Lcn2*, and *Tnf)* by the NF-κB activation cocktail and their suppression by the Notch agonist RIN1 in astrocytes (Supplementary Fig. S10a, Supplementary Table S6). In parallel, Notch target gene expression was found to be upregulated by RIN1, an effect which was suppressed upon NF-κB activation (Supplementary Fig. S10b, Supplementary Table S6).

Importantly, similar transcriptional responses were observed in the activated non-astrocytic flow-through fraction, showing a RIN1-mediated reduction in inflammatory and SASP-associated gene expression (Supplementary Fig. S9a), accompanied by significant downregulation of immune- and NF-κB-related pathways (Supplementary Fig. S9b). In addition, GSEA further revealed negative enrichment of CT-containing genes and SASP genes lacking CT motifs following Notch activation (Supplementary Fig. S9c, d). Similar results were obtained in primary mixed glia cultures (Supplementary Fig. S10c, d, Supplementary Table S7) confirming that pharmaceutical Notch activation is capable to counteract elevated SASP gene expression.

To further validate these findings, C8-D1A astrocytes were co-stimulated with TNF-α and the Notch agonist RIN1, where RIN1 suppressed the TNF-α-induced phosphorylation of IκBα and p65, although degradation of IκBα was not affected (Supplementary Fig. S11). Together, these findings demonstrate that pharmacological Notch activation is able to broadly suppresses the NF-κB-driven gene expression and SASP development in primary astrocytes and non-astrocytic cells, which however is specifically effective in the modulation of CT-containing genes.

## Discussion

In the course of brain inflammaging, reactive astrocytes, microglia, and oligodendrocytes are critically involved in SASP development thereby promoting the evolution of neurodegenerative conditions. Increasing evidence highlights IKK/NF-κB signaling as a central driver of SASP expression and neuroinflammation [11, 40], whereas Notch signaling has been implicated in maintaining brain homeostasis and modulating intercellular communication during aging [9, 41]. Through juxtacrine signaling, Notch coordinates cell-to-cell interactions alongside SASP exposure, facilitating the transmission of senescence through gap junctions [10]. However, the molecular basis by how Notch interfaces with NF-κB signaling to coordinate SASP gene expression in the aging brain has remained insufficiently understood. In this study, we show that brain aging is characterized by an increase in NF-κB signaling which is interconnected with a simultaneous decline in Notch signaling. We could identify a multilevel crosstalk between both pathways, in which activated Notch physically interacts and interferes with key components of the NF-κB pathway. In addition, competition for DNA-binding at shared promoter elements allows differential regulation of SASP gene expression during brain aging.

We also found a strong involvement of astrocytic IKK/NF-κB signaling in the coordination and regulation of the neuroimmune landscape, observing profound changes in infiltration and the myeloid transcriptome of young IKK2-CA^GFAP^ animals which resemble the aging-associated CNS immune phenotype. These includes a significant enrichment in pathways of innate immunity and interferon signaling [42], as well as expressing of a DAM-like gene signature in CD11b^+^ myeloid cells [33–35, 36] among others.

Our group previously found that RBPJ acts as a potent repressor of NF-κB2 (p100/p52) transcription, based on the direct binding to a sequence motif within the p100 promoter that overlaps with a κB element. Moreover, RBPJ binding motifs overlapping with κB elements were identified in the promoters of different human and mouse genes such as *Hes1, Il6, Ifnb1*, and *Igk* [43].These findings prompted us to hypothesize that composite DNA elements with overlapping or adjacent RBPJ and NF-κB motifs function as crosstalk (CT)-sites that are able to integrate signals from both pathways. Supporting this idea, we identified RBPJ/NF-κB-CT sites specifically enriched in such genes associated with SASP and brain aging. These sites also exhibit a high degree of evolutionary conservation, highlighting their regulatory importance in the aging brain. Thus, CT-sites may function as key regulatory hubs, where the balance between NICD-mediated Notch signaling and NF-κB activation determines to which extent target genes are transcriptionally repressed or activated in a context-dependent manner. Integrating *in silico* analyses with reporter assays, we observed differential responses of these CT-sites to NICD versus RelA. Notably, the CT-sites derived from murine *Hes1* and *Il6* promoters exhibited dual responsiveness to both Notch and NF-κB signaling. However, co-expression of NICD with RelA led to a marked suppression of transcriptional activity through these sites, indicating an inhibitory crosstalk at shared promoter elements in which activated Notch signaling counteracts active NF-κB.

Previous studies have shown that RBPJ specifically binds to a DNA region within the IL6 promoter that overlaps with a κB site [44, 45]. These studies support a model in which RBPJ acts both as a repressor of basal *Il6* expression in unstimulated cells and as a modulator of NF-κB accessibility particularly under early or suboptimal stimulation conditions [46]. Our findings extend this model by demonstrating that, upon Notch activation, NICD enhances RBPJ-mediated repression of NF-κB-driven *Il6* transcription through a shared CT-site.

In addition to transcriptional interference at the genomic DNA level, we further demonstrated selective interaction between NICD and the canonical NF-κB subunits RelA and cRel. We hypothesize that the formation of a NICD-RelA complex or “signalosome” may interfere with the nuclear transactivation function of NF-κB. Here, NICD may bind to the Rel homology domain (RHD) and transactivation domain (TAD) regions of RelA, thereby interfering with the RHD-mediated function of sequence-specific DNA binding and dimerization, but may also affects interaction with inhibitory proteins [47]. Specific Co-IP experiments and fine mapping with truncated domain constructs are needed to precisely define the relevant interaction interfaces. A recent study using NIH/3T3 cells, determined that the NICD is able to bind to truncated forms of RelA containing either the RHD (aa 1–300) or the TAD (aa 401–549). Conversely, the ankyrin (ANK) repeat domain of NICD was identified to interact with RelA [48]. This type of interaction does not appear to impede the association of NICD with RBPJ, which occurs mainly at the RAM domain. However, the functional outcome appears to diverge-cooperative in some previous reports but suppressive in our study depending on the cell type, signaling states, and model system. Consistent with our findings, one study supports a repressive role for Notch in NF-κB signaling in Jurkat T cells. The N-terminal portion of NICD (aa 1773–1881) was shown to bind the p50 subunit and block p50/RelA heterodimer binding to κB sites, thereby repressing NF-κB-mediated gene transcription [49]. In studies involving colorectal cancer [50] and glioblastoma cells [51], it promotes a synergistic effect between Notch and NF-κB signaling.

Information on the interplay between NICD and components of IKK complex remains limited, and the specificity and functional implications of such interactions within the brain are still poorly defined. Here, we demonstrate that NICD preferentially interacts with IKK2. This interaction depends on the kinase activity IKK2 and the phosphorylation of S177 and S181 in the activation loop, indicating that NICD specifically recognizes the activated form of IKK2. Accordingly, recent findings in macrophages showed that NICD interacts with IKK2 depending on their complete serine/threonine kinase catalytic (STKc) region and needs the RBP-J associated molecule (RAM) or ANK domain of NICD [48]. Functionally, we found that NICD binding does interfere with downstream signaling (e.g., p65 and IκB phosphorylation) and regular subcellular localization of IKK2 and IKK1, thereby overall inhibiting NF-κB activation. Activation of IKK/NF-κB signaling is primarily dependent on a cytosolic kinase complex formed by IKK1, IKK2, and NEMO subunits. In addition, nuclear IKK, which can be induced by onco-proteins such as mutant BRAF and KRAS or by DNA-damaging agents was reported to exhibit additional functions in tumour evolution [52]. So far it remains open, whether and how the elevated nuclear localisation of IKK1 and IKK2 under conditions of activated Notch signaling does functionally participate in Notch/NF-κB crosstalk signaling. Furthermore, novel phosphorylated substrates have been attributed to IKK1 and IKK2, which are able to expand the perspectives on the biological activities of these kinases [53].

Our data also suggest that IKK2-bound NICD may get impaired in recruitment of transcriptional co-activators necessary for target gene expression. Importantly, we validated NICD–IKK2 interaction in the brain tissue from IKK2-CA^GFAP^ mice while it failed in transgenic mice expressing kinase inactive IKK2 (IKK2-DN), providing direct *in vivo* evidence for NICD/activated IKK2 association. Together, these reciprocal interactions provide a mechanistic framework supporting the view that the increase of inflammatory NF-κB activity upon aging is interconnected with the concurrent decline in Notch signaling.

Interestingly, treatment of primary astrocytes and non-astrocyte cells from wild-type animals with the Notch agonist RIN-1 [39, 54], resulted in the upregulation of Notch target genes, while simultaneously SASP associated genes were downregulated even when these genes were induced by the NF-κB activation cocktail (Fig. 10 and Supplementary Fig. S8–S10).

In summary, our findings identify Notch as a negative modulator of NF-κB signaling and reveal a multi-tiered mechanism by which Notch signaling restrains NF-κB-mediated SASP gene expression: (i) by competing for shared promoter elements [55]; (ii) by interfering with RelA at both cytoplasmic signaling and nuclear transcriptional levels; and (iii) by inhibiting upstream NF-κB activation through IKK2. This integrated repression may play a critical role in limiting inflammatory and SASP-related gene expression during brain aging. Ultimately, targeting Notch/NF-κB crosstalk may offer a promising therapeutic avenue for mitigating brain inflammaging and the progression of neurodegenerative disorders.

## Supplementary Material

gkag1110_Supplemental_Files

## Data Availability

All data are available within the article and its online supplementary data. RNAseq data (GSE318392; GSE347232) and ChIPseq data (GSE314541) are available online.

## Data sharing statement

Supporting data, including methods, figures, and tables, may be found in a data supplement available with the online version of this article. RNAseq data (GSE318392; GSE347232) and ChIPseq data (GSE314541) are available online; for additional original data, please contact [bernd.baumann@uni-ulm.de or franz.oswald@uni-ulm.de].
