# Supplementary material for: Notch/NF-κB crosstalk signaling controls SASP gene expression during brain aging
Source: Nucleic Acids Res. 2026 Nov 5;54(20):gkag1110. doi: 10.1093/nar/gkag1110 (PMC13736121; doi:10.1093/nar/gkag1110)
Supplement: Supplementary file 1 [file gkag1110_supplemental_files.zip › 20260914_Revised_Supplementray materials .pdf]

**Supplementary Materials for:**

# Notch/NF-κB crosstalk signaling controls SASP gene expression during brain aging

Qingwen Yang[1,2]*, Hina Zarrin[1]*, Tabea Hein[3], Burak Yildiz[1], Vivien Prex[3], Konstantinos Siontas[3], Tilman Borggrefe[4], Benedetto Daniele Giaimo[4], Tobias Friedrich[4], M. Lienhard Schmitz[4], Thomas Wirth[3], Franz Oswald[1#] and Bernd Baumann[3#]

[1]University Hospital Ulm, Department of Internal Medicine I, Albert-Einstein-Allee 23, 89081 Ulm, Germany.

[2]Department of Neurology, The Affiliated Suzhou Hospital of Nanjing Medical University, Suzhou 215002, China.

[3]University of Ulm, Institute for Physiological Chemistry, Albert-Einstein-Allee 11, 89081 Ulm, Germany.

[4]Justus-Liebig-University Giessen, Institute of Biochemistry, Friedrichstrasse 24, 35392 Giessen, Germany.

*QY and HZ contributed equally to this work

#Correspondence: Bernd Baumann, University of Ulm, Institute for Physiological Chemistry, Albert-Einstein-Allee 11, 89081 Ulm, Germany; e-mail: bernd.baumann@uni-ulm.de; phone +49 731/50-22898; fax: +49 731/50-22892

#Correspondence: Franz Oswald, University Hospital Ulm, Department of Internal Medicine I, Albert-Einstein-Allee 23, 89081 Ulm, Germany; e-mail: franz.oswald@uni-ulm.de; phone: +49 731/500-44544, fax: +49 731/500-44502

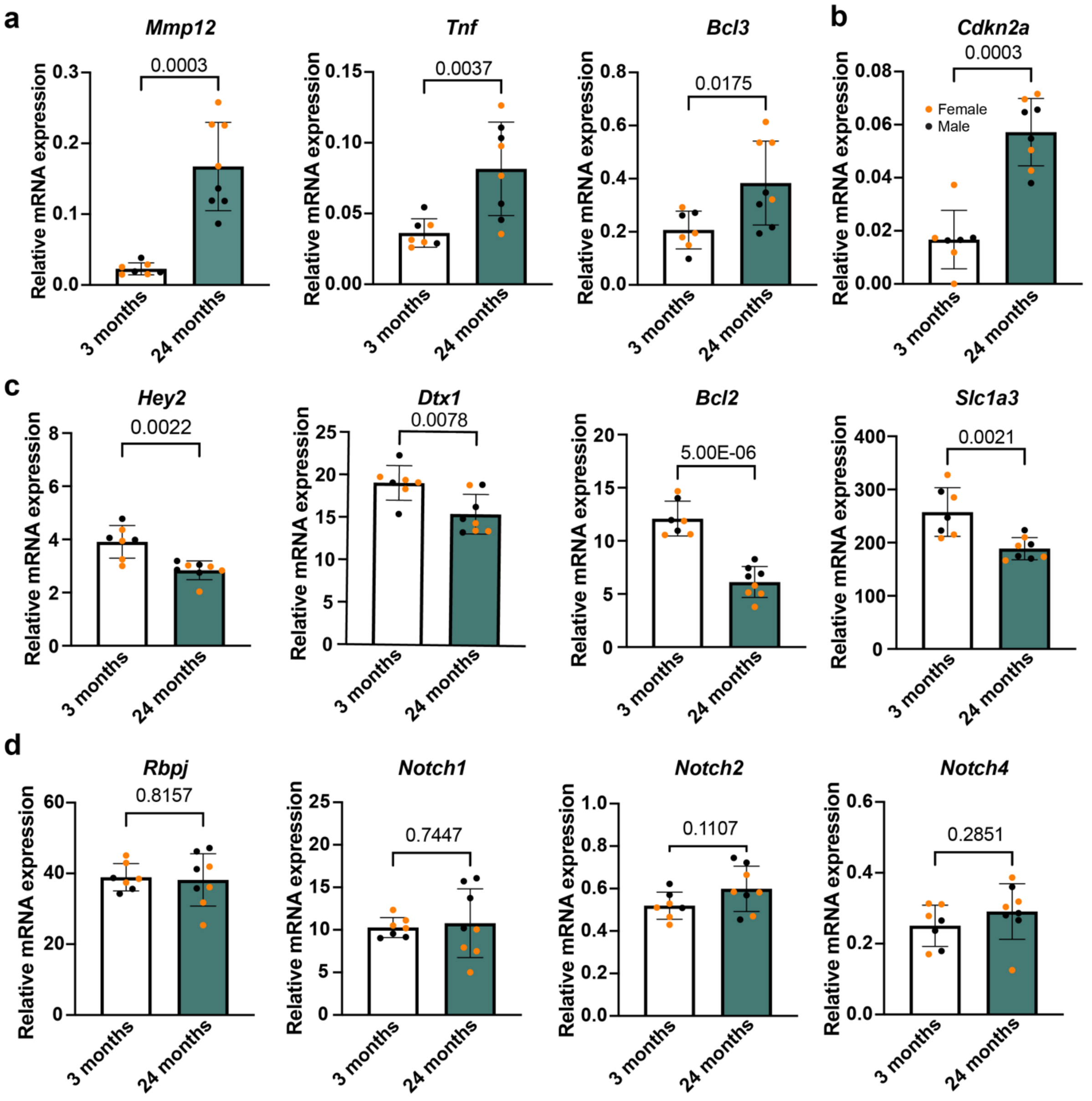


**Supplementary Figure 1. Extended transcriptional profiling of senescence, NF-κB target genes, and Notch pathway components in young and aged mouse brain.** RT–qPCR analysis was performed on cortical tissue from wild-type young (3 months, n=7) and aged (24 months, n=8) mice (C57BL/6J). (**a**) Additional NF-κB target/SASP genes (*Mmp12*, *Tnf*, and *Bcl3*) display elevated expression in aged mice. (**b**) *Cdkn2a* mRNA levels are increased in aged brains, consistent with enhanced cellular senescence. (**c**) Expression of further Notch target genes (*Hey2*, *Dtx1*, *Bcl2*, and *Slc1a3*) is significantly reduced in aged mouse cortex. (**d**) Core Notch pathway components (*Rbpj*, *Notch1*, *Notch2*, and *Notch4*) show no substantial changes in expression with aging. Data was normalized to *Gusb*. Data are shown as mean ± SD. Statistical analysis: unpaired two-tailed Student's t-test or Mann-Whitney test.

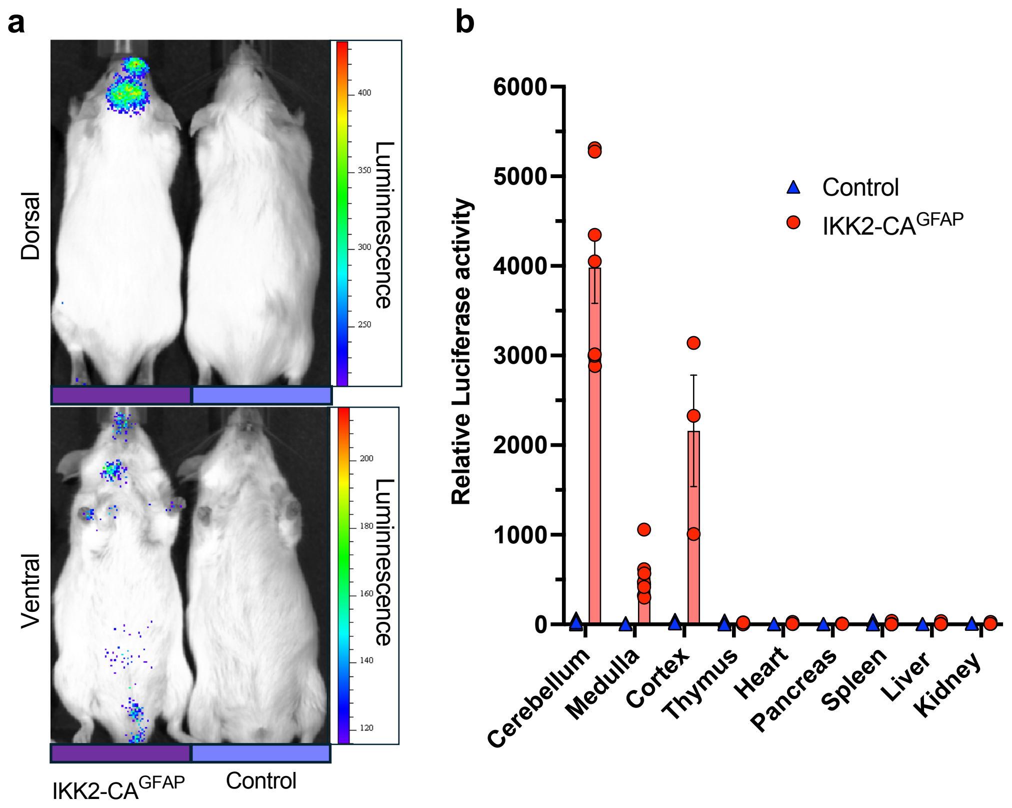


**Supplementary Figure 2. Brain-specific activation of the transgene in IKK2-CA$^{GFAP}$ mice.** Determination of reporter gene activity (**a**) *in vivo* as well as in (**b**) protein extracts of brain regions (cerebellum, medulla, cortex) and various peripheral organs (thymus, heart, pancreas, spleen, liver, kidney) (in RLU/µg] confirms brain-restricted transgene expression in IKK2-CA$^{GFAP}$ animals as compared to control littermates.

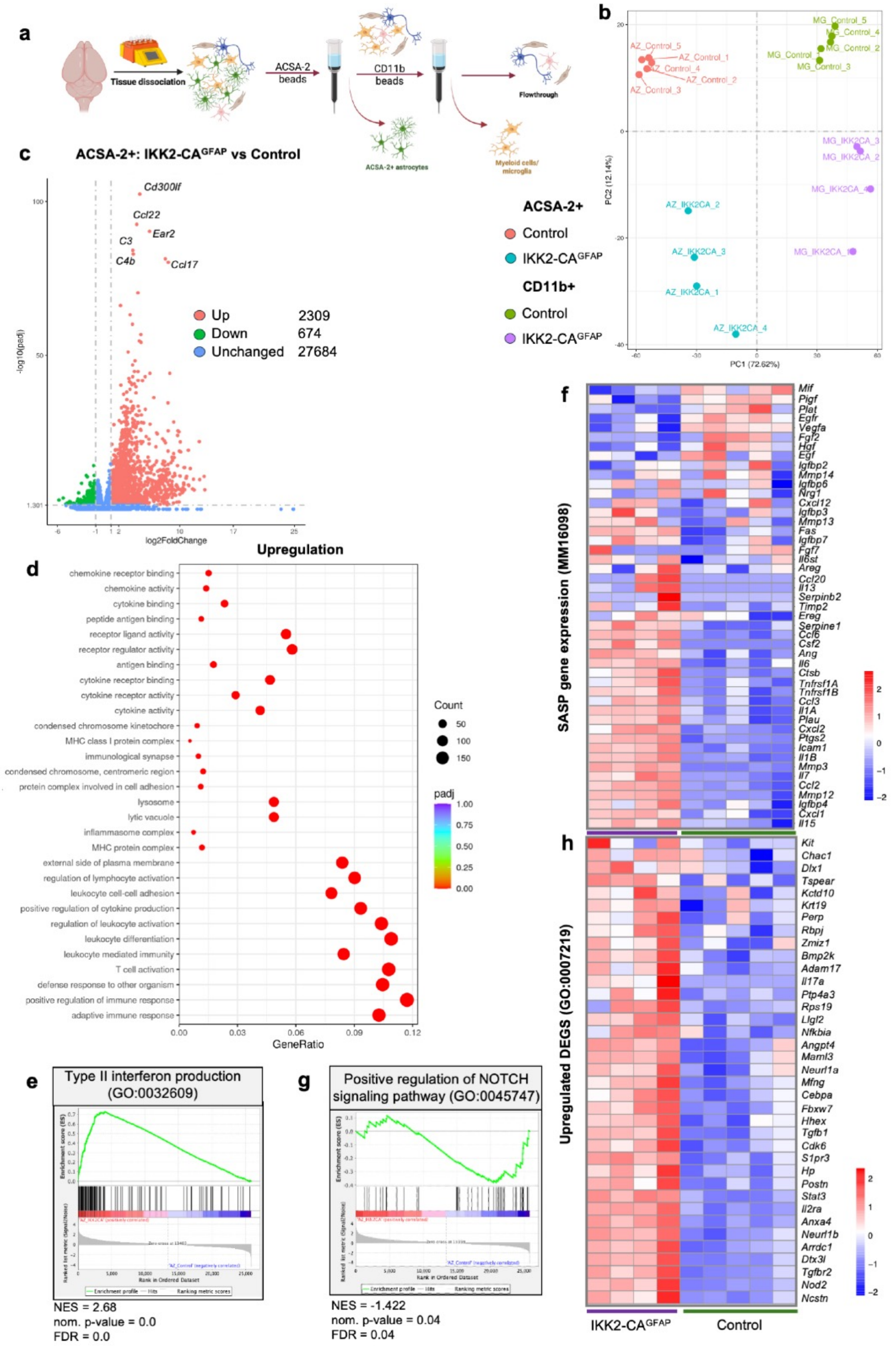


**Supplementary Figure 3. Astrocytic NF-κB activation induces polarization to a pro-inflammatory and immune-reactive transcriptomic signature.**

(**a**) Schematic workflow of the MACS purification protocol to isolate ACSA-2+ and CD11b+

cells from cortical tissue of 5-month-old control and IKK2-CAGFAP (GoF) for subsequent RNA sequencing. Image created with BioRender.com. (**b**) Principal component analysis shows formation of distinct clusters of ACSA-2+ and CD11b+ cells in control and IKK2-CA conditions. (**c**) Volcano plot of differentially expressed genes in GoF astrocytes in comparison to the control group. (**d**) Enrichment chart of the top-upregulated GO terms (biological process, molecular function, cellular component) in GoF astrocytes as compared to the control cohort. (**e**) Gene set-based GSEA (NES = 2.68, FDR = 0.0, nominal p-value = 0.0) of the GO:BP term 'Type II interferon production' (GO:0032609). (**f**) Heatmap depicting expression of assorted SASP genes (gene set: MM16098) in control and IKK2-CAGFAP astrocytes (log2 normalization). (**g**) Gene set-based GSEA (FDR = 0.04, NES = -1.42, nominal p-value = 0.04) of expression of genes found in the GO term 'Positive regulation of NOTCH signaling pathway' (GO:0045747). (**h**) Heatmap depicting NOTCH pathway genes (GO:0007219) upregulated in GoF astrocytes. Statistics: DESeq2 RNA sequencing analysis two-sided Wald's test), |log2FoldChange| > 1, padj-value < 0.05. FDR correction of p-values (padj) by multiple comparisons test. Over- or underrepresentation of gene sets (GSEA analysis) identified by a gene set-based permutation test and subsequent multiple hypothesis testing.

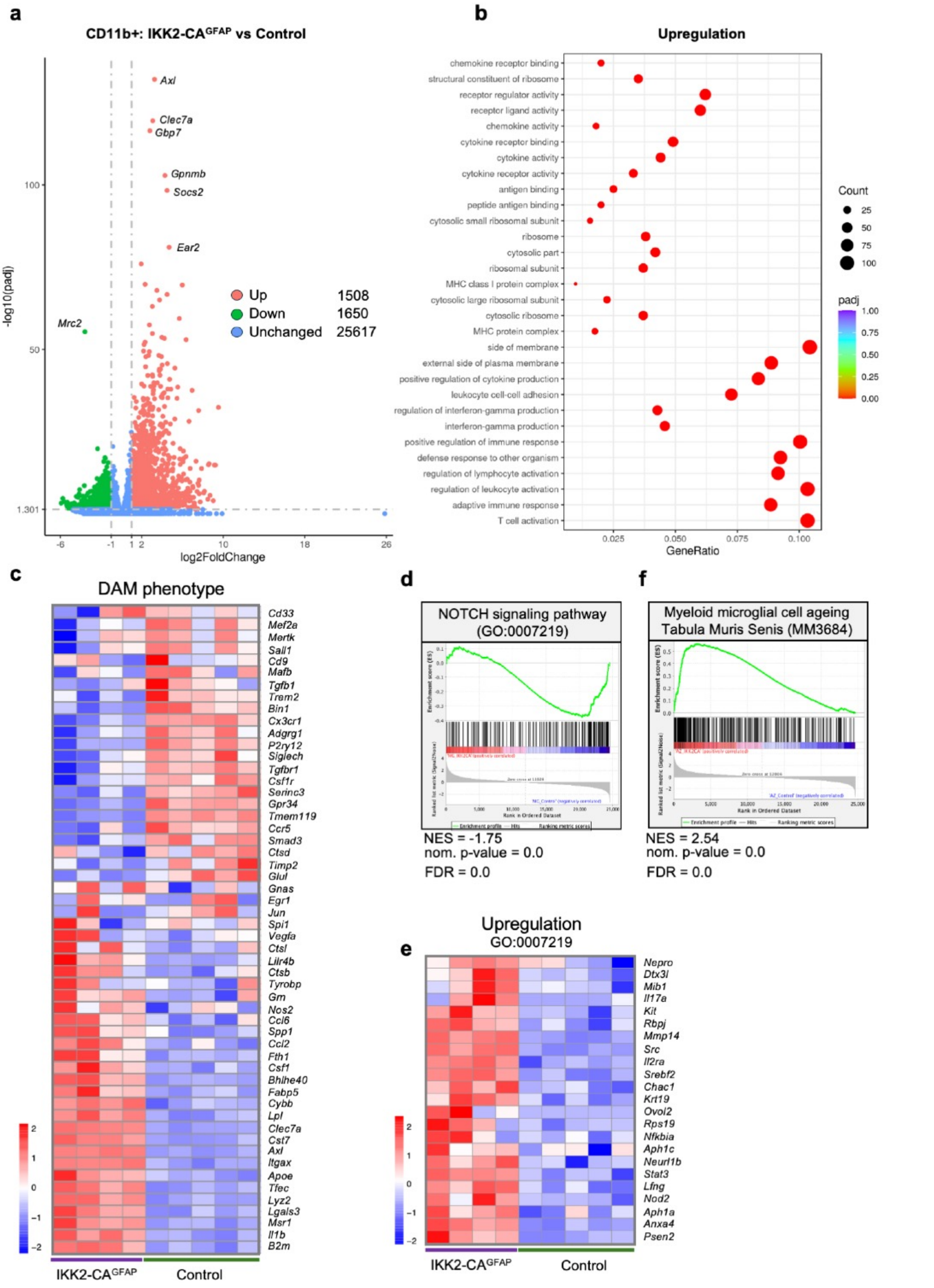


**Supplementary Figure 4. Activation of NF-κB in astrocytes induces neuroinflammatory signature in CD11b$^{+}$ myeloid cells by paracrine communication.** (**a**) Volcano plot of differentially expressed genes in GoF CD11b$^{+}$ cells in comparison to the control group. (**b**)

Enrichment chart of the top-upregulated GO terms (biological process, molecular function, cellular component) in GoF $CD11b^+$ cells as compared to the control cohort. (**c**) Heatmap (log2 normalization) depicting expression of disease-associated microglia (DAM) signature genes. (**d**) Gene-set based GSEA (NES = -1.75, FDR = 0.0, nominal p-value = 0.0) and (**e**) Heatmap of NOTCH pathway genes (GO:0007219) differentially regulated in GoF $CD11b^+$ myeloid cells. (**f**) Gene set-based GSEA (NES = 2.54, FDR = 0.0, nominal p-value = 0.0) of the microglial cell ageing signature (MM3684) published by the Tabula Muris Consortium. Statistics: DESeq2 RNA sequencing analysis two-sided Wald's test), $|log2FoldChange| > 1$, $padj\text{-value} < 0.05$. FDR correction of p-values (padj) by multiple comparisons test.

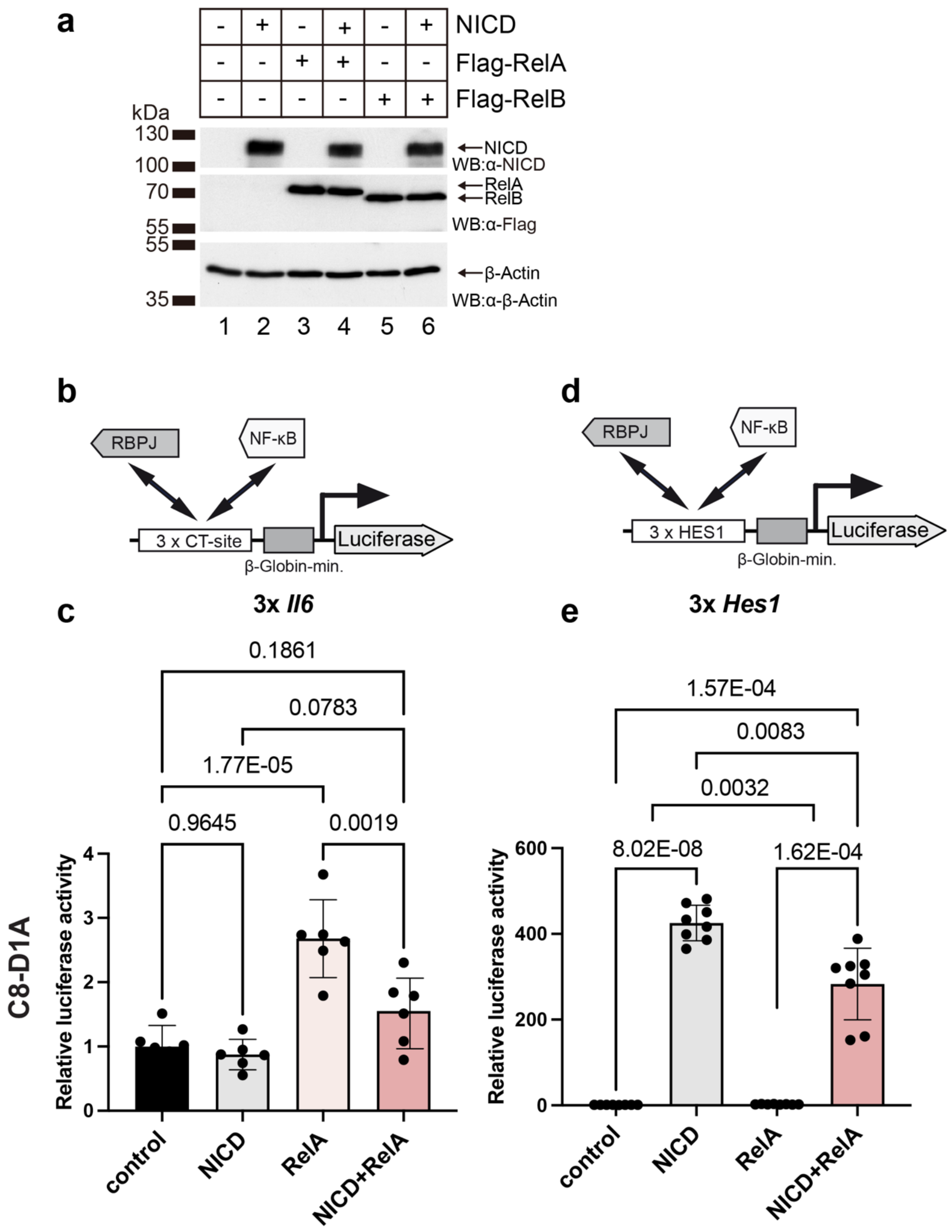


**Supplementary Figure 5. Physical and functional assessment of NICD with NF-κB subunits.** (**a**) Western blot analysis showing the expression of the individual plasmids: NICD (lane 2), RelA (lane 3), and RelB (lane 5), as well as their expression following co-transfection of NICD with RelA (lane 4) and NICD with RelB (lane 6). Co-transfection did not affect the expression levels of the respective plasmids. β-actin was used as a loading control. HeLa cells were transiently co-transfected with 1.2 μg of the NICD plasmid and 1.2 μg of plasmid encoding RelA and RelB. (**b**, **d**) Schematic representation of the 3× CT-site reporter constructs for *Hes1*,

and *Il6*. (**c**, **e**) Quantification of relative luciferase activity from *Il6* (**c**), and *Hes1* (e) in reporters in response to NICD, RelA, or their co-expression in C8-D1A mouse astrocyte cells. Cells were transiently co-transfected with 250 ng of the 3× CT-site reporter construct and 20 ng of expression plasmids encoding NICD, RelA, or both. Data represent mean ± standard deviation from at least three independent experiments, each performed with two technical replicates. All values were normalized to the reporter-only control. Statistical analysis: Brown-Forsythe or Welch's ANOVA, followed by Dunnett's T3 post hoc test was used.

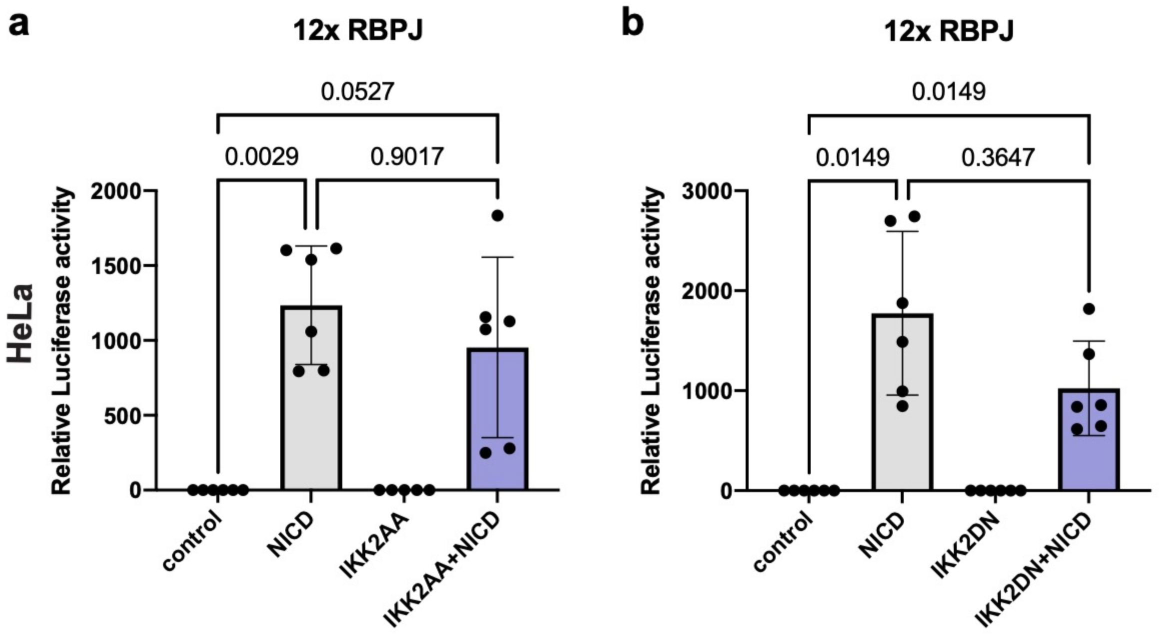


**Supplementary Figure 6**. **Transcriptional activity of Notch with IKK2AA and IKK2DN.** (**a**, **b**) Quantification of relative luciferase activity in HeLa cells transiently co-transfected with 250 ng of 12×RBPJ luciferase reporter and 20 ng NICD, IKK2-AA (**a**), or IKK2-DN (**b**) expression plasmids, alone or in combination. Data represent mean ± standard deviation from at least three independent experiments, each performed with two technical replicates. All values were normalized to the reporter-only control. Statistical analysis: Brown-Forsythe or Welch's ANOVA, followed by Dunnett's T3 post hoc test was used.

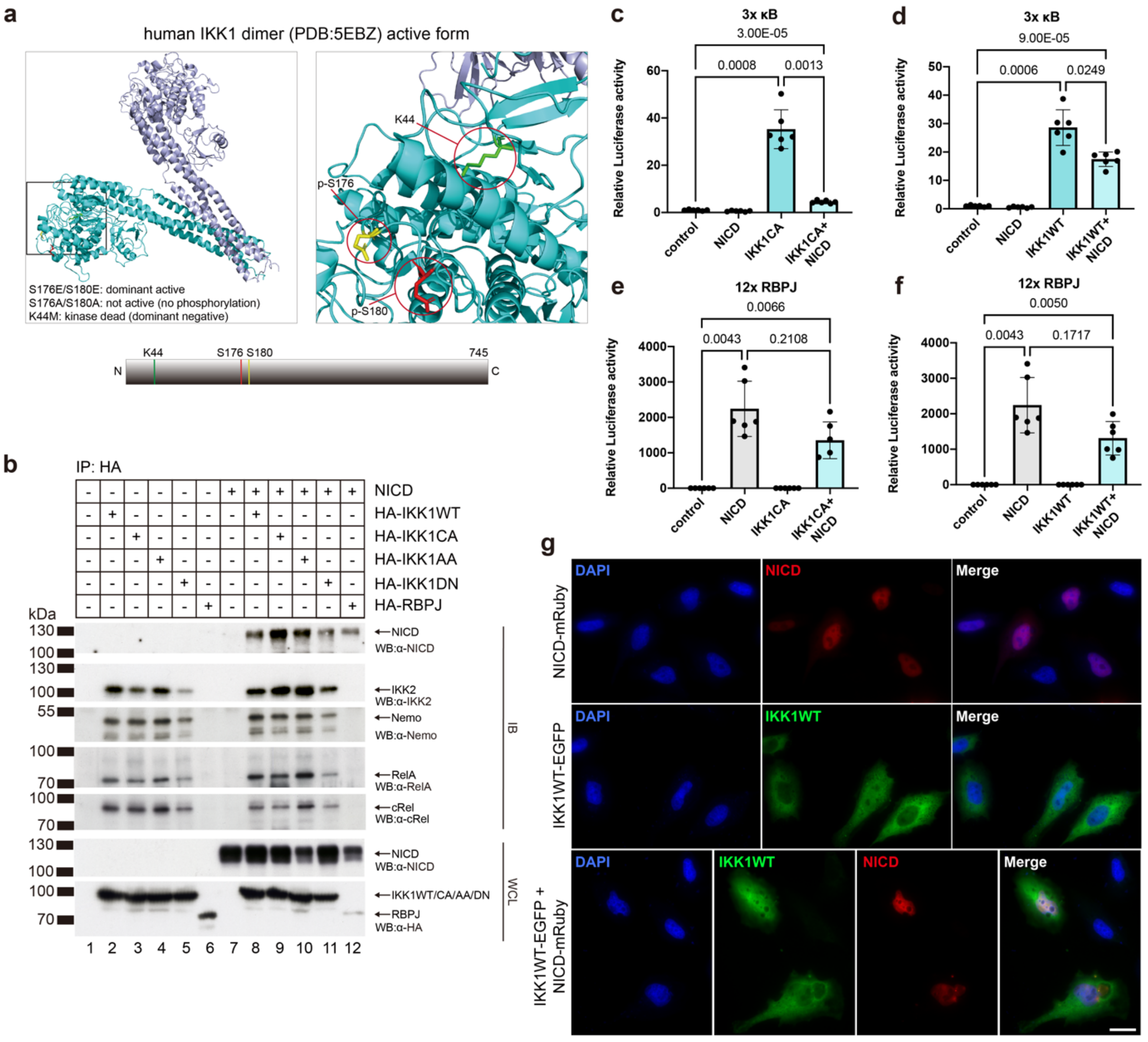


**Supplementary Figure 7. Interaction of NICD with IKK1 is independent of kinase activation and influences NF-κB transcriptional activity.** (**a**) Crystal structure of the human IKK1 dimer (PDB: 5EBZ) with an enlarged view (right panel) of residues K44 (green), S176 (red), and S180 (yellow), corresponding to the DN (K44M), CA (S176E/S180E), and AA (S176A/S180A) forms. A schematic diagram (bottom panel) shows the domain organization and relative mutation positions within the full-length IKK1 protein. (**b**) Co-IP analysis of NICD interactions with functional IKK1 mutants and associated signaling components. HEK293 cells were co-transfected with 10 µg of the indicated expression plasmids encoding HA-tagged IKK1 variants (WT, CA, AA, DN) with or without NICD for 24 h. Protein complexes were immunoprecipitated using anti-HA antibody and protein A beads. HA-RBPJ co-transfected with NICD was included as a positive control. A total of 20 µL of immunoprecipitated samples and 10 µg of WCL were analyzed by SDS-PAGE and immunoblotted with antibodies against NICD (Abcam Limited, Cat. No ab128076), HA, IKK2, NEMO, RelA, and cRel. The top panel shows co-immunoprecipitated NICD (~130 kDa). Subsequent panels display the presence of endogenous IKK2 (~100 kDa), NEMO (~50 kDa), RelA (~70 kDa), and cRel (~70-100 kDa) in the immunoprecipitates. The lower panels confirm expression of the respective proteins in

WCLs. Specific immunoreactive bands are indicated by arrows. (–) and (+) indicate the absence or presence of the corresponding expression constructs. (**c**-**f**) Modulation of Notch and NF-κB transcriptional activity by NICD and IKK1. HeLa cells were transiently co-transfected with 250 ng of either the 3× κB (**c**, **d**) or 12× RBPJ (**e**, **f**) luciferase reporter and 20 ng NICD, IKK2-CA, IKK2-WT expression plasmids, alone or in combination. Data represent mean ± standard deviation from at least three independent experiments, each performed with two technical replicates. All values were normalized to the reporter-only control. Statistical analysis: Brown-Forsythe or Welch's ANOVA, followed by Dunnett's T3 post hoc test. (**g**) Subcellular co-localization of NICD and IKK1. HeLa cells were transfected with 200 ng of NICD-mRuby and IKK1-WT-EGFP, either individually or together. Nuclei were counterstained with DAPI (left column). Fluorescent signals from mRuby or EGFP are shown in the middle column, and merged images are presented in the right column. NICD was localized in the nucleus, while IKK1-WT was distributed in both nucleus and cytoplasm. Co-expression revealed prominent nuclear co-localization of NICD with IKK1-WT. Images were acquired using a fluorescence microscope (60× objective). Scale bars:10 μm.

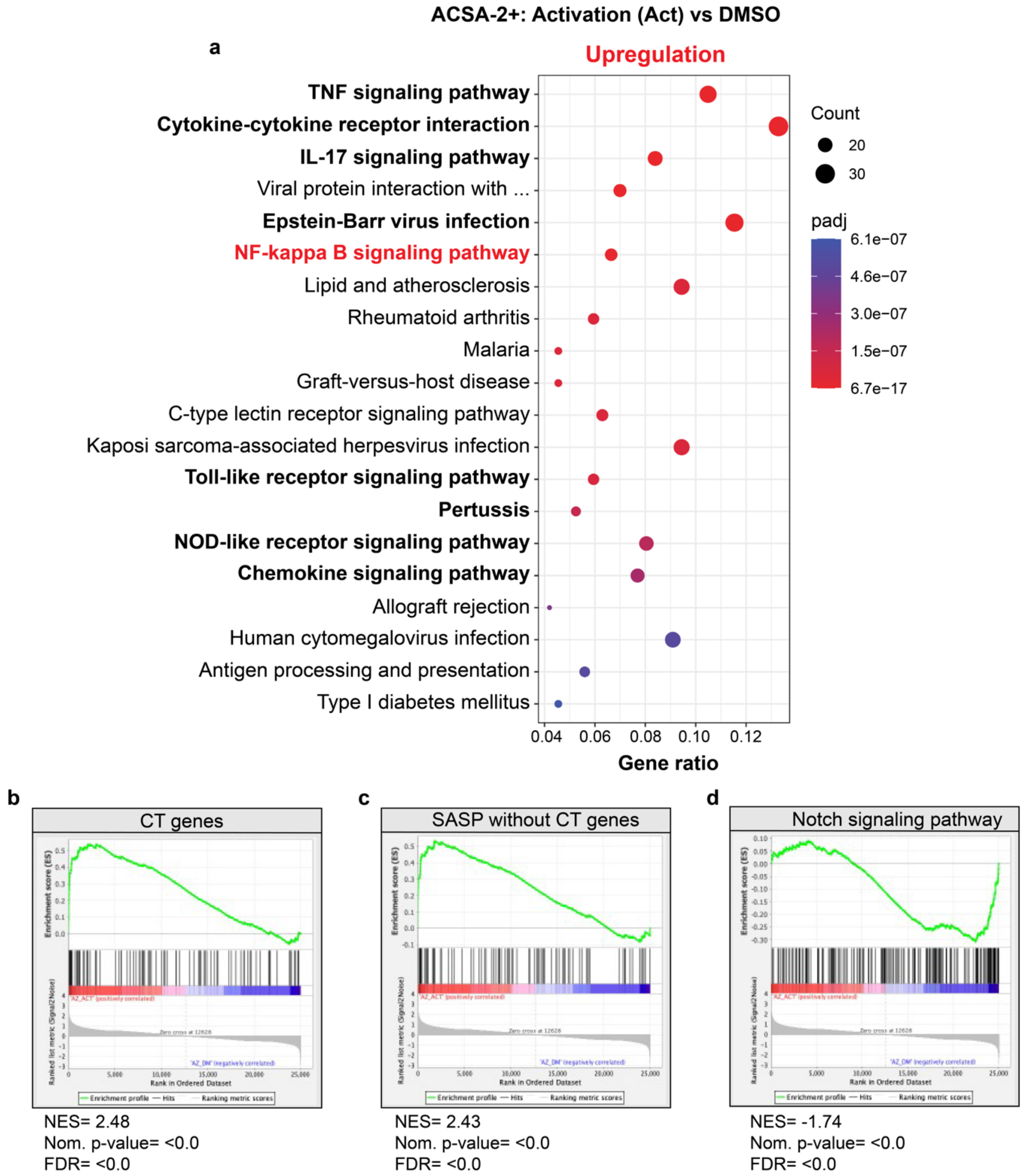


**Supplementary Figure 8. Validation of cell activation cocktail in ACSA-2+ sorted astrocytes.** (**a**) KEGG enrichment analysis showed significant upregulation of pro-inflammatory and NF-κB signaling associated terms upon activation cocktail treatment in astrocytes. Bold and highlighted KEGG terms mark pathways associated with NF-κB signaling. GSEA of activated astrocytes revealed a significant upregulation in (**b**) CT and (**c**) SASP gene expression, while the (**d**) GO term „Notch signaling pathway“ is markedly downregulated. Analysis of RNA sequencing by DESeq2 (two-sided Wald’s test), |log2FoldChange| > 1, p-value < 0.05. FDR correction of p-values (padj) by multiple comparisons test. Over- or underrepresentation of gene sets (GSEA analysis) identified by a gene set-based permutation test and subsequent multiple hypothesis testing.

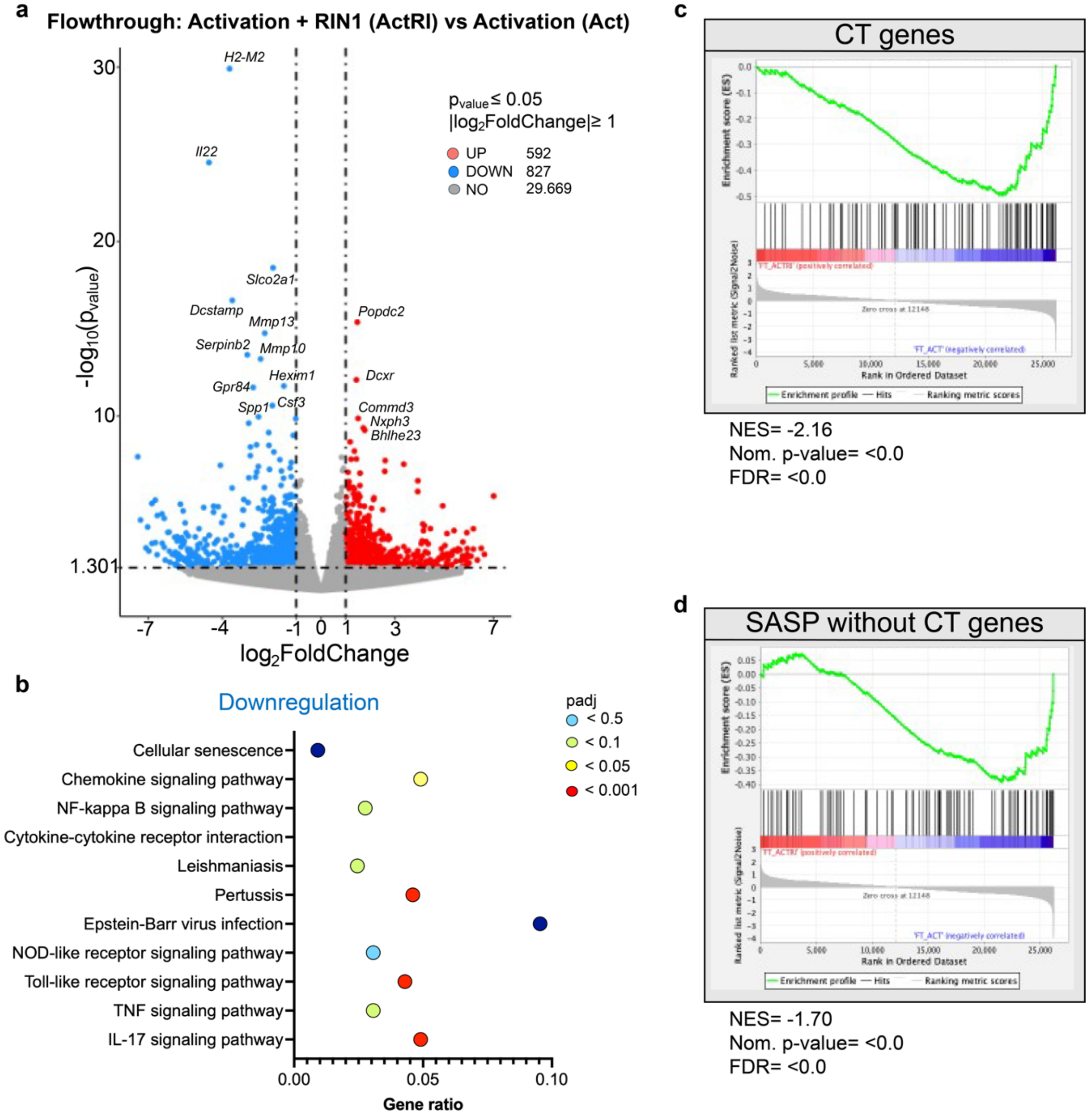


**Supplementary Figure 9. NF-κB activation interferes with NOTCH signaling whereas pharmaceutical Notch activation attenuates NF-κB-driven SASP gene expression.** Treatment of the activated flowthrough cells (mixed non- astrocytic cells) with RIN1 orchestrates a significant downregulation of (**a**) volcano plot)) inflammatory/SASP gene expression and (**b**) KEGG analysis demonstrates a similar downregulation in terms related to immune/NF-κB signaling as indicated. GSEA further confirms a substantial decrease in expression of (**c**) CT and (**d**) SASP genes without genes containing CT motifs as indicated. Analysis of RNA sequencing by DESeq2 (two-sided Wald's test), |log2FoldChange| > 1, p-value < 0.05. FDR correction of p-values (padj) by multiple comparisons test. Over- or underrepresentation of gene sets (GSEA analysis) identified by a gene set-based permutation test and subsequent multiple hypothesis testing.

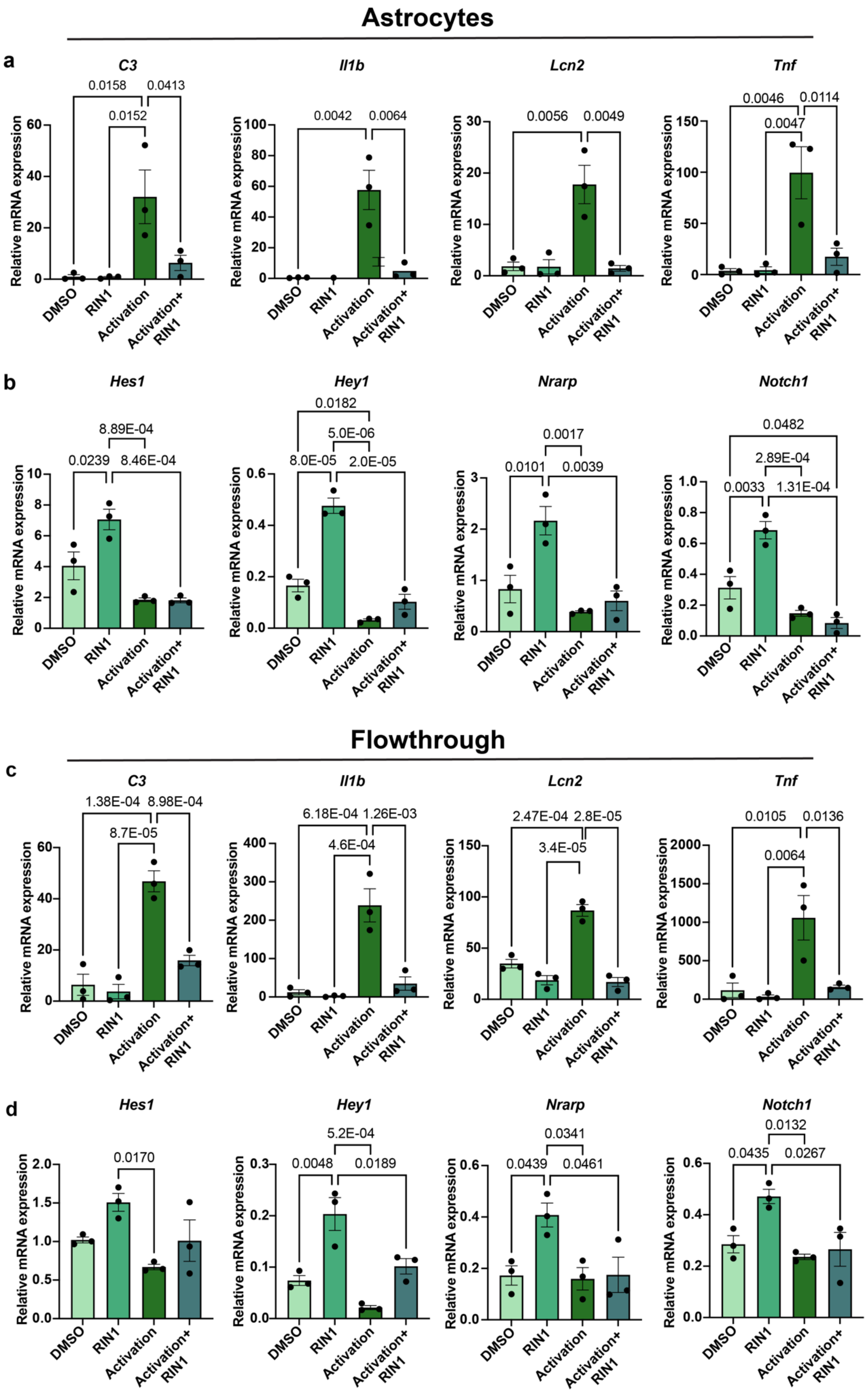


**Supplementary Figure 10. Pharmaceutical Notch activation attenuates NF-κB-driven SASP gene expression.** (**a, c**) qRT-PCR analysis of representative SASP genes (*C3, Il1b,*

*Lcn2, Tnf*) from 14 – 16 weeks old wild-type mice (C57BL/6J) in the purified astrocytes (**a**) and in the purified mixed non- astrocytic cells (**c**). NF-κB-cocktail activated expression is repressed after the treatment of cells with Notch agonist, RIN1. For *Il1b*, 2 datapoints are not detectable. (**b, d**) Notch signaling components (*Hes1*, *Hey1*, *Nrarp*, and *Notch1*) get activated after RIN1 treatment and repressed by induced NF-κB signaling in the purified astrocytes (**b**) and in the purified mixed non- astrocytic cells (**d**). Data was normalized to *Hprt1*. Data are shown as mean ± SD. Statistical analysis: Welch's ANOVA test; n=3.

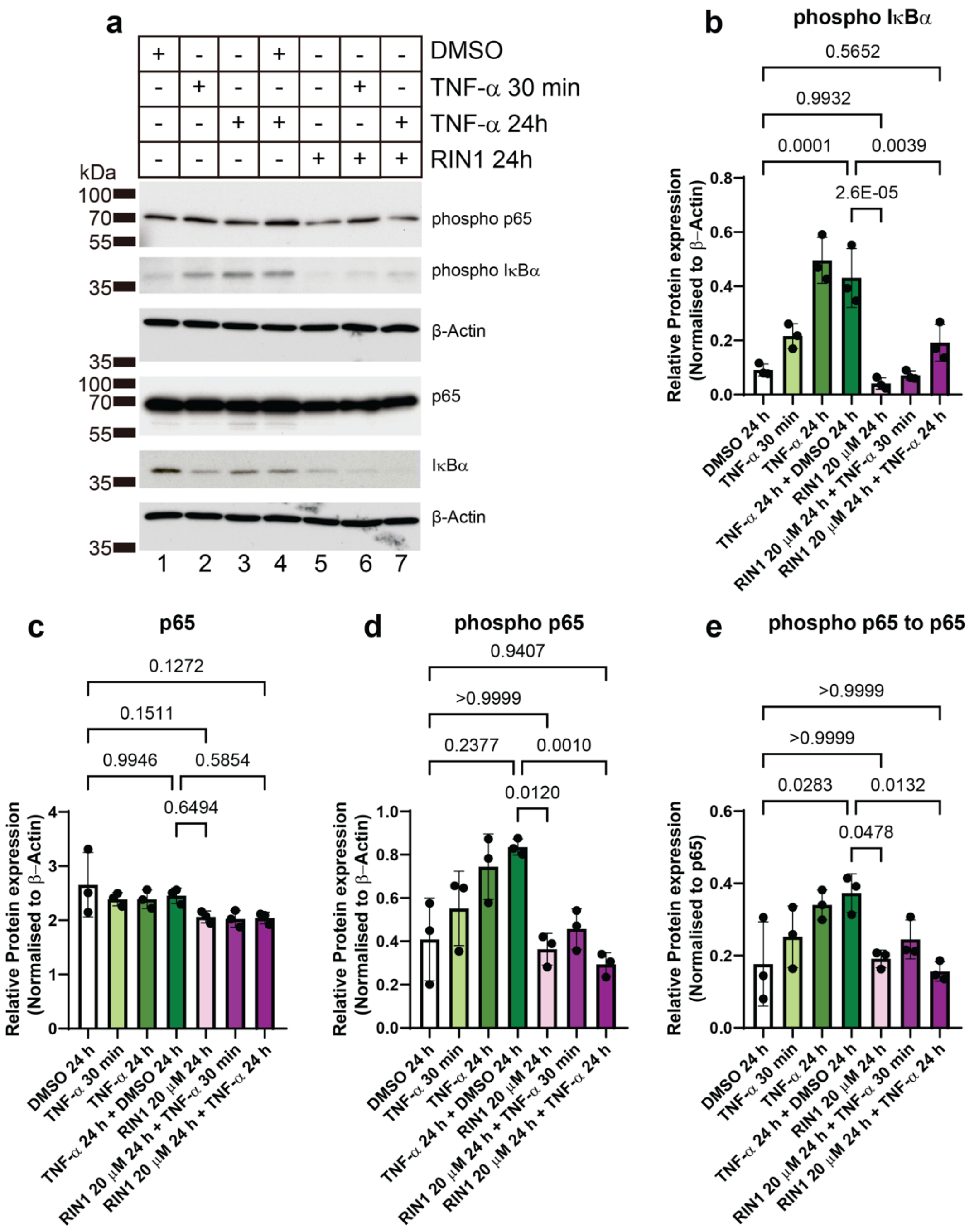


**Supplementary Figure 11. Notch agonist RIN1 suppresses TNF-induced NF-κB signalling in C8-D1A mouse astrocyte cells.** (**a**) Representative western blot images. (**b-e**) Quantification of the expression levels of phospho IκBα (**b**), p65 (**c**), phospho p65 (**d**), phospho p65 to p65 ratio (**e**) following RIN1 and TNF-α stimulation. RIN1 suppressed the phosphorylation of IκBα and p65, including in the presence of TNF-α stimulation. Protein expression levels were normalized to the β-actin loading control. Cells were treated with the Notch agonist RIN1 (24 h), TNF-α (50ng/ml) either for 30 min or 24 h, or a combination of both, with DMSO as a negative control. Statistical analysis: normalized data were analyzed using Ordinary one-way ANOVA followed by Šidák post hoc test; for unequal variances, Brown-Forsythe or Welch's ANOVA with Dunnett's T3 post hoc test was applied. (-) indicates the

absence and (+) denotes the presence of corresponding treatment. Blots representative for 3 biological replicates.

**Supplementary Tables 1, 2, 3 and 4 are given as separate Excel-(.xlsx) files**

**Supplementary Table 5: Comparison of CT site analysis of genes associated with brain and other organs (heart, liver and lung)**

| | Brain | Other organs |
|---|---|---|
| **Analysed genes** | **131** | **59** |
| **Crosstalk** | **78.6** | **33.9** |
| Only RBP-J | 0.8 | 1.6 |
| Only RelA/NF-κB1 | 13.7 | 13.6 |
| RBP-J & RelA/NF-κB1 | 64.1 | 18.6 |
| **No Crosstalk** | **17.6** | **40.7** |
| Only RBP-J | 0.0 | 1.6 |
| Only RelA/NF-κB1 | 2.3 | 8.5 |
| RBP-J & RelA/NF-κB1 | 15.3 | 30.5 |
| **No Occupancy** | **3.8** | **25.4** |

**Supplementary Table 6: Differential expression of genes from RNA seq data in astrocytes**

| ***C3*** | | | | | |
|---|---|---|---|---|---|
| **Group** | **Norm. expression** | | **Fold Change** | **pvalue** | **padj** |
| ACT vs DMSO | 4198.15 | 703.66 | 5.97 | 3.49E-07 | 0.00013 |
| RIN1 vs DMSO | 322.56 | 647.85 | 0.498 | 0.103 | 0.999 |
| ACTRIN vs ACT | 448.47 | 4276.14 | 0.105 | 5.55E-26 | 1.12E-21 |
| ***Il1b*** | | | | | |
| **Group** | **Norm. expression** | | **Fold Change** | **pvalue** | **padj** |
| ACT vs DMSO | 6309.17 | 115.86 | 54.4 | 4.53E-08 | 2.64E-05 |
| RIN1 vs DMSO | 57.98 | 106.25 | 0.545 | 0.465 | 0.999 |
| ACTRIN vs ACT | 433.10 | 6408.93 | 0.068 | 9,58E-08 | 5.39E-05 |
| ***Lcn2*** | | | | | |
| **Group** | **Norm. expression** | | **Fold Change** | **pvalue** | **padj** |
| ACT vs DMSO | 9634.03 | 951.57 | 10.12 | 0.0399 | 0.285 |
| RIN1 vs DMSO | 462.11 | 874.60 | 0.528 | 0.486 | 0.999 |
| ACTRIN vs ACT | 939.47 | 9819.89 | 0.096 | 1.24E-06 | 0.0004 |
| ***Tnf*** | | | | | |
| **Group** | **Norm. expression** | | **Fold Change** | **pvalue** | **padj** |
| ACT vs DMSO | 3172.50 | 360.15 | 8.81 | 0.006 | 0.105 |

| | | | | | |
|---|---|---|---|---|---|
| RIN1 vs DMSO | 94.62 | 329.74 | 0.287 | 0.122 | 0.999 |
| ACTRIN vs ACT | 856.54 | 3220.28 | 0.266 | 9.58E-08 | 5.39E-05 |
| ***Hes1*** | | | | | |
| **Group** | **Norm. expression** | | **Fold Change** | **pvalue** | **padj** |
| ACT vs DMSO | 890.10 | 1378.46 | 0.646 | 0.032 | 0.255 |
| RIN1 vs DMSO | 1034.88 | 1268.04 | 0.816 | 0.404 | 0.999 |
| ACTRIN vs ACT | 450.33 | 904.49 | 0.498 | 0.0208 | 0.324 |
| ***Hey1*** | | | | | |
| **Group** | **Norm. expression** | | **Fold Change** | **pvalue** | **padj** |
| ACT vs DMSO | 672.33 | 1848.33 | 0.364 | 1.64E-06 | 0.0004 |
| RIN1 vs DMSO | 3805.95 | 1698.58 | 2.24 | 0.0019 | 0.438 |
| ACTRIN vs ACT | 1579.18 | 683.39 | 2.31 | 5.54E-05 | 0.0095 |
| ***Nrarp*** | | | | | |
| **Group** | **Norm. expression** | | **Fold Change** | **pvalue** | **padj** |
| ACT vs DMSO | 47.69 | 153.82 | 0.311 | 0.152 | 1 |
| RIN1 vs DMSO | 88.72 | 142.26 | 0.624 | 0.384 | 0.999 |
| ACTRIN vs ACT | 99.08 | 48.76 | 2.03 | 0.386 | 0.891 |
| ***Notch1*** | | | | | |
| **Group** | **Norm. expression** | | **Fold Change** | **pvalue** | **padj** |
| ACT vs DMSO | 3963.86 | 5760.89 | 0.688 | 0.0398 | 0.285 |
| RIN1 vs DMSO | 7622.01 | 5295.61 | 1.44 | 0.071 | 0.999 |
| ACTRIN vs ACT | 4696.64 | 4029.35 | 1.17 | 0.274 | 0.817 |

**Supplementary Table 7: Differential expression of genes from RNA seq data in flowthrough cells (mixed non- astrocytic cells)**

| ***C3*** | | | | | |
|---|---|---|---|---|---|
| **Group** | **Norm. expression** | | **Fold Change** | **pvalue** | **padj** |
| ACT vs DMSO | 6416.71 | 3895.23 | 1.65 | 0.142 | 0.999 |
| RIN1 vs DMSO | 2104.71 | 3705.71 | 0.568 | 0.157 | 0.863 |
| ACTRIN vs ACT | 2072.80 | 6354.36 | 0.326 | 0.00017 | 0.0156 |
| ***Il1b*** | | | | | |
| **Group** | **Norm. expression** | | **Fold Change** | **pvalue** | **padj** |
| ACT vs DMSO | 42601 | 4439.59 | 9.60 | 0.0032 | 0.236 |

| | | | | | |
|---|---|---|---|---|---|
| RIN1 vs DMSO | 1538.67 | 4240.51 | 0.363 | 0.0720 | 0.712 |
| ACTRIN vs ACT | 9654.33 | 42199.36 | 0.229 | 0.00024 | 0.0199 |
| ***Lcn2*** | | | | | |
| **Group** | **Norm. expression** | | **Fold Change** | **pvalue** | **padj** |
| ACT vs DMSO | 40687.47 | 20200.37 | 2.01 | 0.00024 | 0.045 |
| RIN1 vs DMSO | 12186.76 | 19176.64 | 0.635 | 0.107 | 0.793 |
| ACTRIN vs ACT | 17070.88 | 40213.93 | 0.424 | 1.21E-05 | 0.0023 |
| ***Tnf*** | | | | | |
| **Group** | **Norm. expression** | | **Fold Change** | **pvalue** | **padj** |
| ACT vs DMSO | 22922.23 | 4944.33 | 4.64 | 1.39E-05 | 0.0062 |
| RIN1 vs DMSO | 3144.91 | 4700.35 | 0.669 | 0.221 | 0.927 |
| ACTRIN vs ACT | 13243.81 | 22661.58 | 0.584 | 0.219 | 0.676 |
| ***Hes1*** | | | | | |
| **Group** | **Norm. expression** | | **Fold Change** | **pvalue** | **padj** |
| ACT vs DMSO | 1167.93 | 2056.57 | 0.568 | 0.0547 | 0.827 |
| RIN1 vs DMSO | 2080.38 | 1962.07 | 1.060 | 0.859 | 0.999 |
| ACTRIN vs ACT | 1604.82 | 1156.27 | 1.39 | 0.216 | 0.672 |
| ***Hey1*** | | | | | |
| **Group** | **Norm. expression** | | **Fold Change** | **pvalue** | **padj** |
| ACT vs DMSO | 1444.09 | 2664.02 | 0.542 | 0.00067 | 0.0869 |
| RIN1 vs DMSO | 5906.05 | 2532.37 | 2.33 | 0.00019 | 0.0495 |
| ACTRIN vs ACT | 4709.18 | 1430.32 | 3.29 | 4.94E-10 | 5.92E-07 |
| ***Nrarp*** | | | | | |
| **Group** | **Norm. expression** | | **Fold Change** | **pvalue** | **padj** |
| ACT vs DMSO | 291.60 | 793.92 | 0.367 | 0.0153 | 0.5145 |
| RIN1 vs DMSO | 1823.04 | 755.43 | 2.41 | 0.0693 | 0.7041 |
| ACTRIN vs ACT | 779.11 | 288.02 | 2.70 | 0.0148 | 0.2162 |
| ***Notch1*** | | | | | |
| **Group** | **Norm. expression** | | **Fold Change** | **pvalue** | **padj** |
| ACT vs DMSO | 12431.28 | 14735.92 | 0.844 | 0.457 | 0.999 |
| RIN1 vs DMSO | 18976.12 | 14036.30 | 1.35 | 0.321 | 0.968 |
| ACTRIN vs ACT | 11619.72 | 12290.82 | 0.945 | 0.809 | 0.9601 |

**Supplementary Table 8: sequence containing CT site for reporter constructs**

| | |
|---|---|
| SPF_mmHes1 | AAGCTTGCATGCCTGCAGGTC<u>CGTTACTGTGGGAAAGAAAGTTTGGGAAGTTTCACACCGTTACTGTGGGAAAGAAAGTTTGGGAAGTTTCACACCGTTACTGTGGGAAAGAAAGTTTGGGAAGTTTCACAC</u>GATATCACTGTATGCATC |

| | |
|---|---|
| mmIl6 | AAGCTTGCATGCCTACCGGTCTATCAAATGTGGGATTTTCCCATGAGTCTCATATCAAATGTGGGATTTTCCCATGAGTCTCATATCAAATGTGGGATTTTCCCATGAGTCTCAGATCCACTGTATGCATC |
| mmLcn2 | AAGCTTGCATGCCTGCAGGACCAGAGGGACATTCCCAGGGCTTTGGAACCAGAGGGACATTCCCAGGGCTTTGGAACCAGAGGGACATTCCCAGGGCTTTGGAGGATCCACTGTATGCAT |
| mmIfnb1 | AAGCTTGCATGCCTGCAGGTCGACTCTAGAGAACTGAAAGTGGGAAATTCCTCTGAGGCAGAACTGAAAGTGGGAAATTCCTCTGAGGCAGAACTGAAAGTGGGAAATTCCTCTGAGGCAGGATCCACTGTATGCATC |
| mmCdr1 | AAGCTTGCATGCCTGCAGGTCCCGAGTCTTCCCAGAAATTCCCGAGTCTTCCCAGAAATTCCCGAGTCTTCCCAGAAATAGATCCACTGTATGCAT |
| mmIgκ_enhancer | AAGCTTGCATGCCTGCAGGTCCTCAACAGAGGGGACTTTCCGAGAGGCCATCTCAACAGAGGGGACTTTCCGAGAGGCCATCTCAACAGAGGGGACTTTCCGAGAGGCCATGATCCACTGTATGCATC |
| mmTnip1 | AAGCTTGCATGCCTGCAGGCCACGCCCCTGGGAAAGTCCCCGGACCACGCCCCTGGGAAAGTCCCCGGACCACGCCCCTGGGAAAGTCCCCGGAAGATCTACTGTATGCAT |

HinDIII / NsiI fragments cloned into the reporter constructs, 3x CT-site underlined.

**Supplementary Table 9: List of plasmids cloned and validated in this study**

| Construct | Vector | Insert | Enzyme |
|---|---|---|---|
| pGL3-3xmmSPF_ mmHes1 | pGL3-12xRBP | SPF-mmHes1 in pUC57-BsaI-Free (Biocat) | HinDIII / NsiI |
| pGL3-3xmmIl6 | | mmIl6 in pUC57-BsaI-Free (Biocat) | |
| pGL3-3xLcn2 | | mmLcn2 in pUC57-BsaI-Free (Biocat) | |
| pGL3-3xmmIfnb1 | | mmIfnb1 in pUC57-BsaI-Free (Biocat) | |
| pGL3-3xCdr1 | | mmCdr1 in pUC57-BsaI-Free (Biocat) | |
| pGL3-3xIgκ_enhancer | | mmIgκ_enhancer in pUC57-BsaI-Free (Biocat) | |
| pGL3-3xTnip1 | | mmTnip1 in pUC57-BsaI-Free (Biocat) | |
| pcDNA3-Flag-IKK2WT | pcDNA3-Flag-IKK2CA | pcDNA3-IKK2wt-EosFP(T158H) | BspEI / BamHI |
| pcDNA3-IKK2WT-EGFP | pcDNA3-IKK2wt-EosFP(A69V) | pcDNA-EGFP-ATG | XhoI / XbaI |

**Supplementary Table 10: List and description of plasmids available in the laboratory and used in this study**

| Name | Description |
|---|---|
| 3xκB luci | NF-κB reporter plasmid with 3 tandem κB-motifs in front of a beta-Globin minimal promoter in front of the Luciferase cDNA |
| pcDNA3-3xHA-IKK1-WT | Expression plasmid for human, hemaglutinine-tagged wild type IKK1 |
| pcDNA3-3xHA-IKK1-EE | Expression plasmid for human, hemaglutinine-tagged constitutively active IKK1 (S176E/S180E) |
| pcDNA3-3xHA-IKK1-AA | Expression plasmid for human, hemaglutinine-tagged phosphorylation-deficient IKK1 (S176A/S180A) |

| | |
|---|---|
| pcDNA3-3xHA-IKK1-DN | Expression plasmid for human, hemaglutinine-tagged dominant-negative IKK1 (K44M) |
| pcDNA3-Flag RelA | Expression plasmid for human, Flag-tagged RelA / p65 |
| pcDNA3-Flag RelB | Expression plasmid for human, Flag-tagged RelB |
| pcDNA3-Flag-IKK2-CA | Expression plasmid for human, Flag-tagged constitutively active IKK2 (S177E/S181E) |
| pcDNA3-Flag-IKK2-AA | Expression plasmid for human, Flag-tagged phosphorylation-deficient IKK2 (S177A/S181A) |
| pcDNA3-Flag-IKK2-DN | Expression plasmid for human, Flag-tagged dominant-negative IKK2 (D145N) |
| pcDNA3 RelA | Expression plasmid for human RelA/p65 |
| pcDNA3-p52 | Expression plasmid for human p52 |
| pcDNA3-hup65-YFP | Expression plasmid for human RelA/p65 fused to the YFP cDNA |
| pcDNA3-mNotch1-IC-mRuby | Expression plasmid for murine NICD-1 fused to the mRuby cDNA |
| pcDNA3-Flag3-RBP2N | Expression plasmid for human Flag-tagged RBPJ splice variant RBP2N |
| pcDNA3.1(-)hsNICD | Expression plasmid for human NICD-1 |
| pcDNA3.1(-)hsNICD-EGFP | Expression plasmid for human NICD-1 fused to the EGFP cDNA |
| pcDNA3.1-HA-RBP | Expression plasmid for human, hemaglutinine-tagged RBP |
| pcDNA5 FRT/TO hup65wt | Expression plasmid for human, wild type p65 / RelA |
| pcDNA5 FRT/TO hup65 E39I | Expression plasmid for human, DNA-binding-defective p65 (RelA-E39I) |
| PEF puro hup65(wt) HA | Expression plasmid for human, hemaglutinine-tagged wild type p65 / RelA |
| PEF puro hup65(E39I) HA | Expression plasmid for human, hemaglutinine-tagged DNA-binding-defective p65 (RelA-E39I) |
| pGL3-12xRBPJ | Reporter plasmid containing twelve RBPJ binding sites, a Beta-Globin promoter in front of the Luciferase cDNA |

**Supplementary Table 11: List of antibodies used in this study**

| Antibody | Dilution | Species | Company |
|---|---|---|---|
| ACSA-2, APC (REAfinity™) (#130-116-142) REA969\|IH3-18A3 | 1:50 | Mouse | Miltenyi Biotec (Bergisch Gladbach) |
| Anti-GFP [IgG1κ] (clones 7.1 and 13.1) (11814460001) | 1:1000 | Mouse | Merck KGaA (Darmstadt, Germany) |
| Armenian Hamster IgG, FITC (#400906) HTK888 | 1:100 | Armenian Hamster | BioLegend (San Diego, California, USA) |

| | | | |
|---|---|---|---|
| CD11b, BV711(#101242) M1/70 | 1:500 | Human/ Mouse | BioLegend (San Diego, California, USA) |
| CD11b, PE (#130-113-797) M1/70.15.11.5 | 1:100 | Human/ Mouse | Miltenyi Biotec (Bergisch Gladbach) |
| CD45, APC (#103112) 30-F11 | 1:1000 | Mouse | BioLegend (San Diego, California, USA) |
| CD45, BV605 (#103140) 30-F11 | 1:500 | Mouse | BioLegend (San Diego, California, USA) |
| ECL™ Anti-mouse IgG HRP-conjugated (NA931) | 1:5000 | Sheep | Cytiva (Marlborough, Massachusetts, USA) |
| ECL™ Anti-rabbit IgG HRP-conjugated (NA934) | 1:5000 | Donkey | Cytiva (Marlborough, Massachusetts, USA) |
| IgG1, PE (REAfinity™) (#130-113-446) REA293\|IS5-21F5 | 1:50 | Human | Miltenyi Biotec (Bergisch Gladbach) |
| IgG1, PE (REAfinity™) (#130-113-450) REA293\|IS5-21F5 | 1:50 | Human | Miltenyi Biotec (Bergisch Gladbach) |
| IgG2c, PE (#400707) RTK4174; kappa | 1:100 | Rat | BioLegend (San Diego, California, USA) |
| Monoclonal Anti-Flag M2-Affinity Gel (A2220) | 1:10 | Mouse | Sigma-Aldrich Chemie GmbH (Steinheim, Germany) |
| Monoclonal Anti-Flag M5 [IgG1] (F4042) | 1:1500 | Mouse | Sigma-Aldrich Chemie GmbH (Steinheim, Germany) |
| Monoclonal Anti-HA, High Affinity (3F10) [IgG1] (11867423001) | 1:1000 | Rat | Merck KGaA (Darmstadt, Germany) |
| Monoclonal Anti-IKKβ (D30C6) (8943) | 1:100 (IP) 1:1000 (WB) | Rabbit | Cell Signaling Technology (Danvers, Massachusetts, USA) |
| Monoclonal Anti-IKKγ/NEMO [IgG1] (611306) | 1:1000 | Mouse | BD Transduction Laboratories™ (Franklin Lakes, New Jersey, USA) |
| Monoclonal Anti-Notch1 (5B5) [IgG2b] (3447) | 1:50 (IP) | Rat | Cell Signaling Technology (Danvers, Massachusetts, USA) |
| Monoclonal Anti-Notch1 [IgG1, mN1A] (ab128076) | 1:1000 | Mouse | Abcam Limited (Cambridge, UK) |

| | | | |
|---|---|---|---|
| Monoclonal Anti-α-Tublin (T9026) | 1:1000 | Mouse | Sigma-Aldrich Chemie GmbH (Steinheim, Germany) |
| Monoclonal Anti-β-Actin (#A1978) | 1:5000 | Mouse | Sigma-Aldrich Chemie GmbH (Steinheim, Germany) |
| Monoclonal IκBα (44D4) (#4812) | 1:1000 | Rabbit | Cell Signaling Technology (Danvers, Massachusetts, USA) |
| Monoclonal NFκB p65 (D14E12) (#8242) | 1:1000 | Rabbit | Cell Signaling Technology (Danvers, Massachusetts, USA) |
| Monoclonal Phospho- NFκB p65 (Ser536) (93H1) (#3033) | 1:1000 | Rabbit | Cell Signaling Technology (Danvers, Massachusetts, USA) |
| Monoclonal Phospho-IκBα (Ser32) (14D4) (#5209) | 1:200 | Rabbit | Cell Signaling Technology (Danvers, Massachusetts, USA) |
| Mouse TrueBlot® ULTRA: Anti-Mouse IgG HRP (18-8817-30) | 1:5000 | Rat | Rockland Immunochemicals, Inc. (Limerick, Pennsylvania, USA) |
| Notch1 Recombinant Monoclonal Antibody (SJ205) (MA5-32080) | 1:1000 | Rabbit | Thermo Fisher Scientific (Waltham, Massachusetts, USA) |
| O4, PE (REAfinity™) (#130-117-711) REA576\|O4 | 1:50 | Human/ Mouse/ Rat | Miltenyi Biotec (Bergisch Gladbach) |
| Peroxidase AffiniPure Anti-rat IgG HRP-conjugated (112-035-003) | 1:5000 | Goat | Jackson ImmunoResearch Europe Ltd. (Cambridgeshire, United Kingdom) |
| Polyclonal Anti-NFκB p65 (PA5-16545) | 1:500(WB)<br>1:300 (IF) | Rabbit | Thermo Fisher Scientific (Waltham, Massachusetts, USA) |
| Polyclonal Anti-RelB (25027-1-AP) | 1:1000 | Rabbit | Proteintech Germany GmbH (Planegg-Martinsried, Germany) |
| Rabbit TrueBlot® ULTRA: Anti-Rabbit IgG HRP (18-8816-33) | 1:5000 | Mouse | Rockland Immunochemicals, Inc. (Limerick, Pennsylvania, USA) |
| Recombinant Monoclonal Anti-c-Rel [EPR2559(2)](ab133251) | 1:200 (IP)<br>1:1500 (WB) | Rabbit | Abcam Limited (Cambridge, UK) |
